# Reducing soft tissue artefacts through projection of markers and microwave imaging: An exploratory study

**DOI:** 10.1038/s41598-025-89586-w

**Published:** 2025-03-05

**Authors:** Vignesh Radhakrishnan, Martin Robinson, Niccolo M. Fiorentino, Samadhan B. Patil, Adar Pelah

**Affiliations:** 1 School of Physics, Engineering and Technology, University of York, York, UK; 2 Department of Mechanical Engineering, University of Vermont, Vermont, USA; 3 York Biomedical Research Institute, University of York, York, UK; 4 Center for Complex Systems & Brain Sciences, Florida Atlantic University, Boca Raton, USA

**Keywords:** Soft tissue artefacts, Biomechanics, Microwave imaging, Medical research, Engineering

## Abstract

Soft tissue artefacts (STA) are widely considered the most critical source of error in skin-mounted marker-based biomechanics, negatively impacting the clinical usability of skin-mounted marker-based data. Amongst the numerous solutions proposed to ameliorate STA, incorporating true bone movement—acquired using adaptive constraints, projection of markers, or various imaging modalities—has been reported to improve kinematic accuracy. However, efficacy of these proposed solutions reduces for different investigated motions and participants. In this study, we propose two novel marker projection schemes, wherein a cluster of markers are projected onto the bone surface during motion. Additionally, we investigate the feasibility of applying a novel, safe and cost-effective imaging modality—microwave imaging—to detect the location of the bone from the skin surface. Our results indicate that the novel marker projection schemes reduce kinematic errors significantly (by 50%) and improve the quality of computed kinematics (95% correlation to true bone movement). In addition, our results show that microwave imaging was able to detect the bone from the skin surface in both male and female anatomical models of varying body mass index scores and poses. We believe our findings underscore the generalisability and applicability of our proposed solution to reduce STA.

## Introduction

Movement analysis is a widely applied clinical tool used to aid in the diagnosis of musculoskeletal and neurological pathologies, and to quantify the outcomes of rehabilitation or a surgical procedure^1^. Skin-mounted marker-based systems are the clinical gold standard for movement analysis and work by placing reflective (or active) markers on specific landmarks on the human body, the marker’s recorded trajectories are then analysed to evaluate the motion of the body in three planes: sagittal (side), coronal (frontal) and transverse. Joint angle deviations in the three planes are analysed in clinical gait laboratories to diagnose various pathologies and plan surgical and non-surgical interventions. In particular, clinical movement analysis using skin-mounted markers is widely applied in cerebral palsy (CP). CP is a group of lifelong neurological disorders which affect movement and appear in infancy or early childhood. Intoeing gait is one of the most common gait abnormalities in children with CP (present in over 60% of children with CP)^2^and can cause pain and discomfort, as well as severe functional issues^3^. The main treatment for intoeing gait is a surgical procedure, femoral derotation osteotomy, with the decision to perform this surgery dependendent on the joint angles computed during clinical gait analysis using skin-mounted markers^3,4^.

However, the clinical usability of data (joint angles) generated by skin-mounted markers is significantly affected by soft tissue artefacts (STA). STA are discrepancies in bone movement computed using skin-mounted markers when compared with true bone movement, and are caused by the interposition of soft tissues between the skin-mounted markers and the underlying bones, with STA reported to significantly affect joint angles computed in the coronal and transverse plane^5,6^. For example, in CP, one recommended prerequisite for performing femoral derotation is a minimum internal hip rotation of $15^{\circ }$, which is to account for STA-induced inaccuracies in computing joint angles in the transverse plane during clinical gait analysis^7^. Studies^8^ have also reported that the hip internal/external rotation angle had the most error in 3-D gait analysis, which impacts pre-operative planning and the success of femoral derotation osteotomies.

STA are subject-, marker location- and task-specific, with traditional filtering methods ineffective at reducing their deleterious impact. Therefore, STA are considered the most critical source of error in clinical movement analysis^5,9^, with a plethora of solutions having been proposed to mitigate its effects on computed kinematics. However, no single solution has proven to be effective for all analysed motions and for all individuals. In addition, most experimental research undertaken to determine solutions to compensate for STA has used participants with a healthy body mass index (BMI)^10^score, despite evidence of a direct correlation between higher BMI scores and increased magnitudes of STA^11^.

Amongst the numerous solutions proposed to reduce the deleterious effect of STA, studies have indicated that incorporating true bone movement (hereafter referred to as artefact-free bone movement) into kinematic pipelines is the most effective in reducing the effect of STA^12–15^. For example, incorporating adaptive joint boundary conditions computed from artefact-free bone movements—obtained using intracortical pins—in musculoskeletal models resulted in significantly different kinematics when compared with kinematics obtained using conventional joint boundary conditions^12^, with the computed kinematics acquired using adaptive joint boundary conditions found to reduce errors in joint rotations used to identify risk factors for musculoskeletal injuries^16^. Incorporating artefact-free bone movement into kinematic pipelines have even been reported to reduce STA for obese participants. For example, an obesity-specific markerset, which used a digital pointer to identify and track the underlying bony anatomical landmarks, produced similar results to a conventional markerset on non-obese participants, but produced higher fidelity pelvic tilt angles and significantly smaller muscle forces, compared with a conventional markerset, when applied to obese participants^14^.

Additionaly, whilst not directly computing artefact-free bone movement, another marker projection scheme was also proposed to reduce the effects of STA in the upper body during activities of daily living and sports^15^. Skin-mounted markers were projected onto a requested axis of the local coordinate system (LCS) to cancel out the deleterious effect of STA on computed kinematics. For example, the local coordinates of the upper-arm skin-mounted markers ,except those along the longitudinal axis of the segment (axis parallel to the humerus), were set to 0. The results indicated that projection of a subset of markers, or projection of all the markers on the cuff, reduced kinematic errors by 20% when compared with conventional (un-projected) skin-mounted markers. However, the authors also observed that the projection of all skin-mounted markers increased kinematic errors for some investigated motions. This was attributed to the loss of information when setting coordinates equal to 0^15^.

An alternative method of obtaining artefact-free bone movement, often applied to investigate small bone movements which are masked by STA, are imaging modalities such as fluoroscopy, MRI, computed tomography (CT) and ultrasound^17–19^. Two notable studies^13,20^incorporated ultrasound imaging—a safe and cost-effective imaging modality—to compensate for STA. One study proposed an intelligent ultrasound sensor which would be capable of determining the distance between bone and skin during motion and thereby reduce the effects of STA on computed kinematics^20^. Whilst the proposed sensor was tested using in-vivo tests, it has not been validated on tissue-mimicking phantoms nor on humans, with the STA compensation scheme still to be validated^21^. The other study developed a system which combined ultrasound imaging with a skin-mounted marker-based system (CAT & MAUS) to determine and track the underlying greater trochanter anatomical landmark during motion^13,22^. The proposed system was validated in-vivo and in-vitro, with results indicating that the system could achieve errors of less than $1.2^{\circ }$ in estimating the femur position, and with errors of reconstructing the femur shape 1/10th of those obtained using just skin-mounted markers. However, the system is currently only capable of imaging the greater trochanter, with an ultrasound probe required to be held at the surface of the greater trochanter during motion.

The two studies reviewed above leveraged ultrasound imaging to determine the location of the bone^13,20^during motion in order to reduce the effects of STA (by computing artefact-free bone movement). Although ultrasound imaging is a safe and cost-effective imaging modality when compared with MRI, fluoroscopy or CT, it has the following limitations: the need for a probe to be held at the location to be imaged, the need for coupling liquid to improve resolution and the need for a radiologist’s input when images are unclear. Microwave imaging (MI), also a safe (non-ionising), low power (negligible heating effects) and cost-effective imaging modality, is operator-independent with the potential to be applied for imaging any part of the human body^23^, thereby overcoming the above drawbacks of ultrasound imaging.

MI leverages the difference in electrical properties (permittivity and conductivity) between various tissues, and between healthy and diseased tissues, to detect and image the object of interest, and has been extensively applied in breast and brain tumour imaging^24–26^. A typical MI system incorporates an imaging domain, a radio-frequency switch, a vector network analyser and antennas. The antennas are placed rigidly around the imaging domain, with both the antennas and imaging domain immersed in a coupling liquid^27^. A large number of antennas (minimum of 32) or scanning positions (when antennas are rotated around the imaging domain) are generally used, in order to acquire sufficiently large amounts of data to reconstruct the object of interest. The systems are generally static and are of considerable size: an enclosure of radius 22.4 cm and height 44.4 cm was used to image human forearms^28^; a tank of height 8.2 cm and radius 2.7 cm was used to validate a bone phantom^29^.

Therefore, a typical MI system cannot be easily integrated into biomechanical applications which ideally require a system which is portable, wearable and has a small form-factor. Whilst several studies have investigated wearable MI systems to overcome the limitations of a static microwave imaging system^30–32^, none of the above systems were proposed for use in a dynamic application—such as biomechanical applications—and therefore were validated on stationary subjects. Similarly, whilst guidelines were proposed for a wearable microwave imaging system^33^, this study focused on potential improvements which could be achieved in the reconstructed images, and did not elucidate how such a system may be applied in a portable fashion or be applied during motion^33^.

Imaging algorithms, which are applied with MI systems, can be broadly classified into tomographic or quantitative imaging algorithms (which provide both the location of the scatterer and the distribution of electrical properties in the image) and radar-based or qualitative imaging algorithms (which are used to only detect the presence of a strong scatterer)^34–41^. Whilst studies have incorporated quantitative imaging algorithms to determine the electrical properties of the bone in the human forearm^28^,leg^42^and heel^43^, quantitative imaging algorithms remain computationally expensive, require the conversion of scattering parameters to electric field values, and are generally not applied in real-time^27,44^. Comparatively, qualitative imaging algorithms are computationally inexpensive, can be directly applied to scattering parameters and can be applied in real-time. Therefore, in this investigation, qualitative imaging algorithms were leveraged.

The two objectives of this study are: 1) to propose a novel method of reducing STA by projecting markers onto the bone surface and to evaluate its potential to improve kinematic accuracy specifically in hip rotation angles; 2) to investigate the the feasibility of leveraging microwave imaging to determine the location of the bone from the skin surface to enable the above proposed marker projection schemes. For our first objective we validate the efficacy of our novel marker projection schemes—offset-projection and closestPoint-projection—by comparing kinematics computed using our marker projection schemes to kinematics computed using conventional (un-projected) skin-mounted markers and another marker projection scheme wherein the markers are projected onto the longitudinal axis^15^. For our second objective we validate the feasibility of applying microwave imaging in biomechanics by detecting the location of the femur using data collected under the following conditions: no-coupling liquid (direct contact of the antenna with the skin), reduced number of antennas and the need for the system to detect the location of the bone in both a static pose and in a gait mimicking pose. These conditions were chosen to emulate a wearable system^31^.

Through this study, we aim to propose a generalisable and novel STA reduction pipeline and system, which is safe, cost-effective and portable, and which may help reduce errors affecting the usability skin-mounted marker-based systems.

## Methods

### Projection of markers

#### Data

Data used in this study were obtained from the dataset leveraged in studies^45,46^, with permission provided by the authors. The dataset contains skin-mounted marker trajectories and artefact-free trajectories of bony landmarks recorded using dual-fluoroscopy (and presented as virtual dual-fluoroscopy markers, DF) for 11 subjects performing five activities: standing, level walk, incline walk, internal hip rotation to end range of motion and external hip rotation to end range of motion. Reflective markers were placed on the following landmarks: anterior-superior iliac spines (ASIS), posterior-superior iliac spines (PSIS), iliac crests (ILC), lateral epicondyle, greater trochanter and a cluster of four markers on the lateral thigh. Bony landmarks recorded using dual-fluoroscopy (DF) were: ASIS , PSIS, ILC, lateral and medial epicondyles, greater and lesser trochanter. Only the pelvis and thigh of the ipsilateral side were imaged, therefore the dataset only contains trajectories of skin-mounted markers and DF-markers of the pelvis and thigh of the imaged side^45,46^. The dataset also contained setup files for segment scaling and marker registration on OpenSim^47^for all subjects. The data used in this study was collected after obtaining informed signed consent from the participants in accordance with relevant guidelines and regulations, with all experimental protocols approved by University of Utah’s Institutional Review Board^45,46^. Please refer the Acknowledgments section for more details.

#### Musculoskeletal modelling and projection of markers

A publicly available generic rigid-body musculoskeletal model^48^was used in OpenSim^47^. As only the pelvis and femur data were available, other segments (shank, feet, torso) were removed from the generic model. The generic model was scaled to subject-specific dimensions using the DF-markers of the pelvis and femur of the standing trial. Skin-mounted virtual markers and DF-virtual markers were registered to their experimental counterparts using the standing trial. Errors for scaling process (segment scaling and marker registration) were below the guidelines recommended by OpenSim (Root mean square error [RMSE] < 1cm and maximum marker error < 2cm).

Three projection schemes were implemented for the cluster of markers at the thigh:

1. Setting the anterior-posterior (x) and lateral-medial (z) coordinates of each thigh marker in the LCS to 0. This scheme was proposed in paper^15^. This projection is henceforth called Begon-projection.
2. Shifting the entire cluster of thigh markers radially to the bone surface by assigning the the lateral-medial (z) coordinate of the cluster of markers in the LCS to that of bone. The location of the bone in the LCS was determined using the DF-markers. This projection is henceforth called Offset-projection.
3. Projecting each marker of the thigh cluster to the closest point on the bone surface. The bone surface was determined in the LCS and each marker of the cluster was projected to the closest point on the bone surface in the LCS. This projection is henceforth called closestPoint-projection.

The coordinates of the markers in the LCS were determined using the scaled model in OpenSim. The trajectories of the projected markers in the global (lab) coordinate system were computed using the PointKinematics tool in OpenSim wherein the joint angles computed using only the DF-markers were leveraged to calculate the location of each projected marker in the global coordinate system at each time step. These trajectories were then combined with the un-projected skin-mounted marker trajectories of the pelvis to calculate joint angles.

**Fig. 1:**
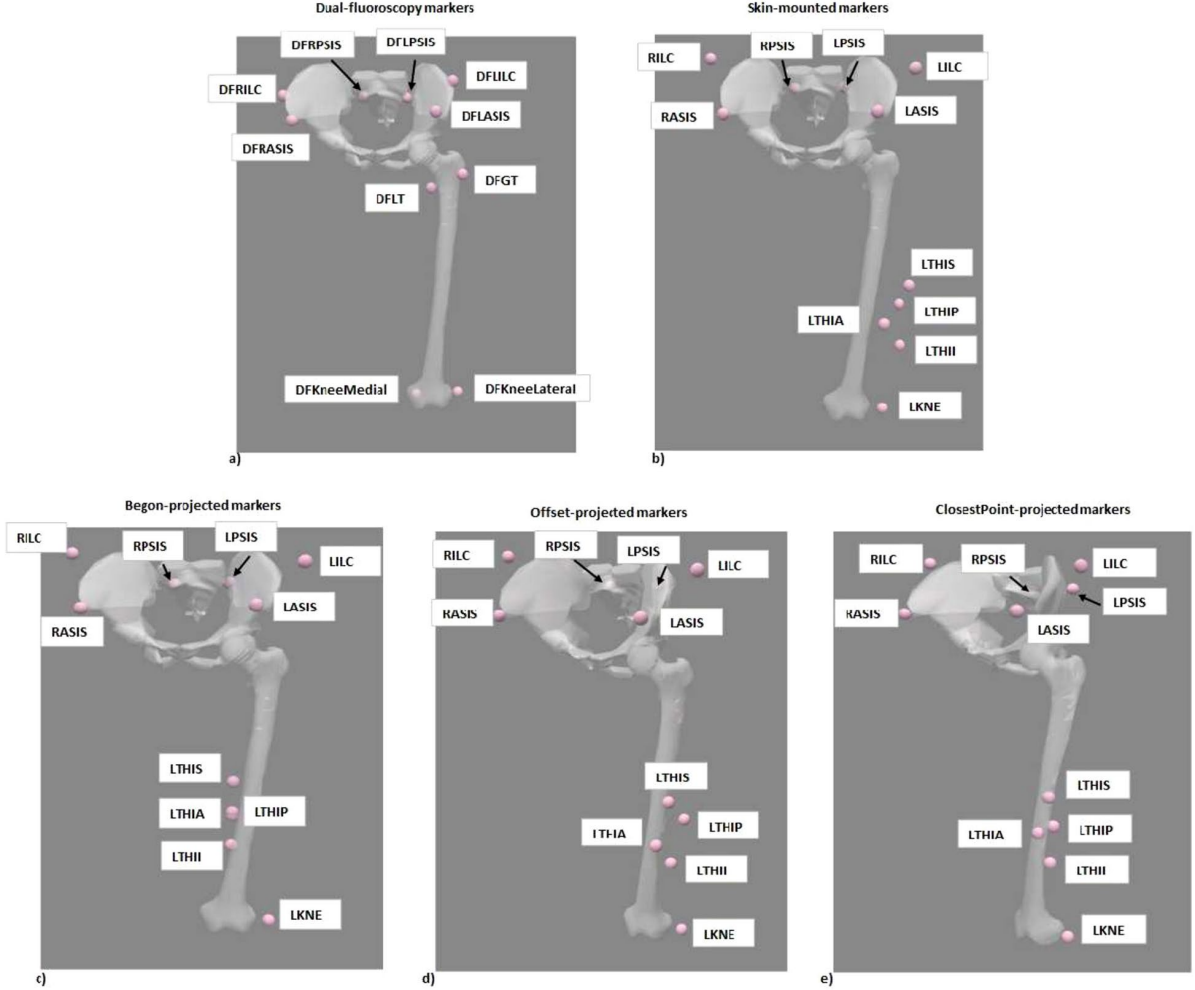
Differing markersets analysed. (**a**) Dual-fluoroscopy markers of the pelvis and thigh. The marker names are DFRASIS: Dual-fluoroscopy based right anterior-superior iliac spine, DFLASIS: Dual-fluoroscopy based left anterior-superior iliac spine, DFRPSIS: Dual-fluoroscopy based right posterior-superior iliac spine, DFLPSIS: Dual-fluoroscopy based left posterior-superior iliac spine, DFRILC: Dual-fluoroscopy based right iliac crest, DFLILC: Dual-fluoroscopy based left iliac crest, DFGT: Dual-fluoroscopy based greater trochanter, DFLT: Dual-fluoroscopy based lower trochanter, DFKneeLateral: Dual-fluoroscopy based lateral epicondyle and DFKneeMedial: Dual-fluoroscopy based medial epicondyle. (**b**) Skin-mounted markers on the pelvis and thigh. (**c**) Begon-projected thigh markers and skin-mounted markers on the pelvis. (**d**) Offset-projected thigh markers and skin-mounted markers on the pelvis. (**e**) closestPoint-projected thigh markers and skin-mounted markers on the pelvis. The skin-mounted markers are RASIS: Right anterior-superior iliac spine, LASIS: Left anterior-superior iliac spine, RPSIS: Right posterior-superior iliac spin, LPSIS: Left posterior-superior iliac spine, RILC: Right iliac crest, LILC: Left iliac crest, LTHIS, LTHI,LTHIA, LTHIP: Left thigh cluster of markers, LKNE: Lateral epicondlyle.

Offset-projected markers have similar movement to conventional (un-projected) skin mounted markers in the anterior-posterior (x) and superior-inferior (y) directions with the lateral-medial movement restricted due to projection. ClosestPoint-projected markers restricts the movement in all three planes compared to conventional (un-projected) skin-mounted markers. The five different markersets: DF-markers, skin-mounted markers, Begon-projected markers, Offset-projected markers and closestPoint-projected markers, are shown in Figure 1.

Hip joint angles and total residual errors (the difference between model-derived marker location and experimental marker location) for level walking, incline walking, internal and external hip rotation were calculated using the inverse kinematic (IK) analysis in OpenSim. Hip joint angles and total residual errors were computed for the following cases:

1. DF-markers were given a weight of 1. All projected and conventional (un-projected) skin-mounted markers were given a weight of 0.
2. Conventional (un-projected) skin-mounted markers were given a weight of 1. All DF-markers and projected markers were given a weight of 0.
3. Begon-projected markers, the conventional (un-projected) skin-mounted lateral epicondyle marker and pelvis conventional (un-projected) skin-mounted markers were given a weight of 1. Conventional (un-projected) skin-mounted markers on the femur, DF-markers and other projected markers were given a weight of 0.
4. Offset-projected markers, the conventional (un-projected) skin-mounted lateral epicondyle marker and pelvis conventional (un-projected) skin-mounted markers were given a weight of 1. Conventional (un-projected) skin-mounted markers on the femur, DF-markers and other projected markers were given a weight of 0.
5. closestPoint-projected markers, the conventional (un-projected) skin-mounted lateral epicondyle marker and pelvis conventional (un-projected) skin-mounted markers were given a weight of 1. Conventional (un-projected) skin-mounted markers on the femur, DF-markers and other projected markers were given a weight of 0.

The hip joint in the generic musculoskeletal model is modelled as a ball joint (only contains 3 degrees of freedom [DoF]). To investigate the effect of projection of markers on different joint models, the above steps were repeated for the hip joint modelled as a 6 DoF joint i.e no joint constraints between the pelvis and femur.

#### Data comparison and statistical analysis

Joint angles computed using just the DF-markers were taken as reference joint kinematics. Cross-correlation coefficients and RMS errors were calculated between joint kinematics computed using just the DF-markers and those computed using conventional (un-projected) skin-mounted markers and conventional (un-projected) skin-mounted markers with projected markers. Paired *t*-tests between joint angle errors acquired using conventional (un-projected) skin-mounted markers and projected markers were performed for all joint angles.

In addition to comparing joint angle errors, residual errors computed using the different approaches (using just DF-markers, conventional (un-projected) skin-mounted markers and different projected markers) were compared. Residual errors are used as a goodness-of-fit metric between the model and the underlying data^49–51^ in the absence of artefact-free bone movement.

All statistics tests were performed in MATLAB. Non-parametric tests were conducted if normality could not be assumed. Normality was tested using the Andersen-darling test in MATLAB.

### Feasibility of microwave imaging

To investigate the feasibility of applying microwave imaging in biomechanical applications, data was collected under specific conditions using models of antennas and humans of different BMI scores. Specifically, to detect the location of the bone from the skin surface in both static and gait mimicking poses, three conditions were applied:

- No coupling liquid was to be used in the system with the antennas making direct contact with the skin. This was to ensure that the system developed could translate to a wearable and portable system, whilst also ensuring there was sufficient coupling between the antennas and the human body (thereby reducing the reflection of electrical field at the air-skin interface)
- The number of antennas was restricted to 8. This was to ensure that the time for data collection is minimal and can record data during different phases of a gait/motion cycle i.e at each data collection instance the tissues encompassed by the antennas can be considered to be static
- The location of the bone from the skin surface should be detected both in static pose and in poses mimicking gait

The antennas and human models used for the investigation are described in the following subsections.

#### Antenna investigated

Various antennas operating at different frequencies have been proposed for microwave imaging. Even amongst microwave imaging studies analysing the dielectric properties of the bone, different antennas have been leveraged: microstrip antennas operating in the frequency range of 1.5- 4.5 GHz were used to monitor bone health^29^, monopole antennas operating in the frequency range of 0.5–3GHz were leveraged to detect variations in bone density due to injuries^43^and dipole antennas operating in the frequency range of 0.8–1.2GHz were used to study human forearms^28^. The frequency ranges were chosen to optimise image resolution and penetration into the human body. Based on the reviewed studies, we have determined the optimal frequency range—for both image resolution and sufficient penetration into the human body—to be between 0.5–3 GHz.

**Fig. 2:**
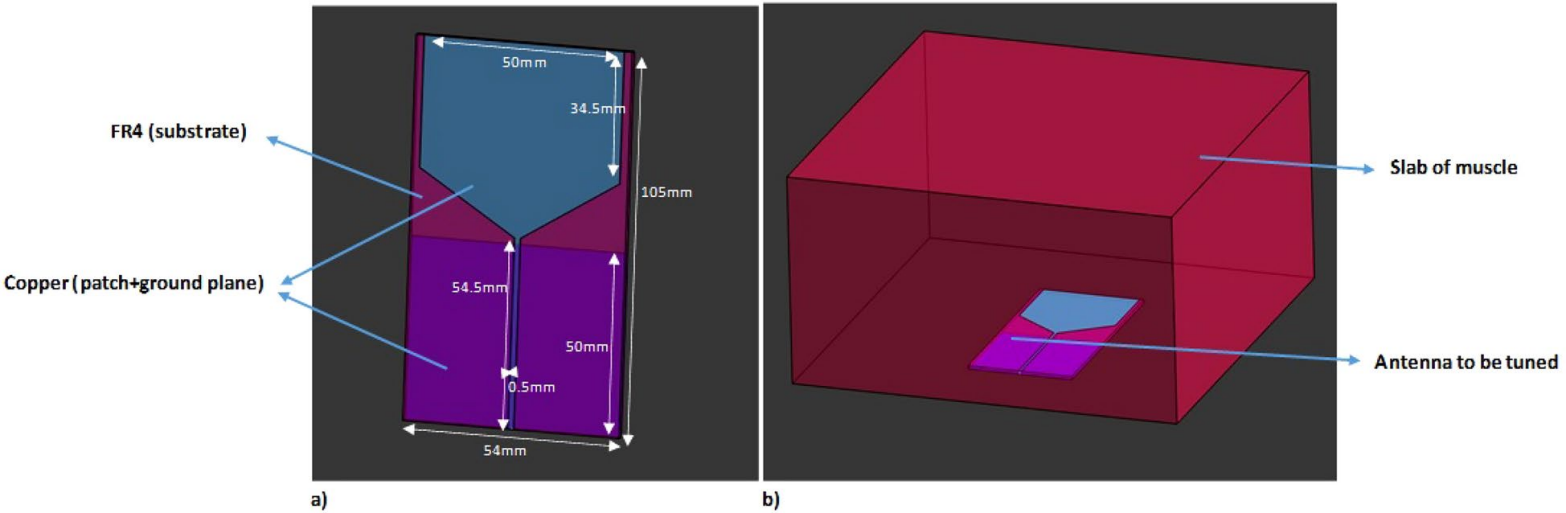
(**a**) The antenna investigated in this study, the modified triangular monopole patch antenna. (**b**) Method to tune the patch antenna by maximising coupling into a slab of muscle.

In our study, we used a triangular patch monopole antenna (Figure 2a) which was proposed for brain tumour detection and was tuned to operate between 1–3GHz when immersed in a coupling liquid^52^. This antenna was further tuned for maximum coupling into the human body in the absence of any coupling liquid (Figure 2b) by parametrically altering the dimensions of the patch and antenna size to determine the optimal shape for maximal coupling into a slab of muscle in Sim4Life^53^.

#### Virtual population models investigated

Four virtual population (ViP) models available in Sim4Life^54,55^were leveraged in this study. Duke, a male anatomical model, and Ella, a female anatomical model, were the baseline models used in the study. Additionally, two morphed models of Ella—where the fat and muscle content were increased to mimic humans with different BMI scores, whilst preserving the internal organ placement and tissue distribution^55^ —were also leveraged. The characteristics of the four anatomical models used in this study are given in Table 1.

**Fig. 3:**
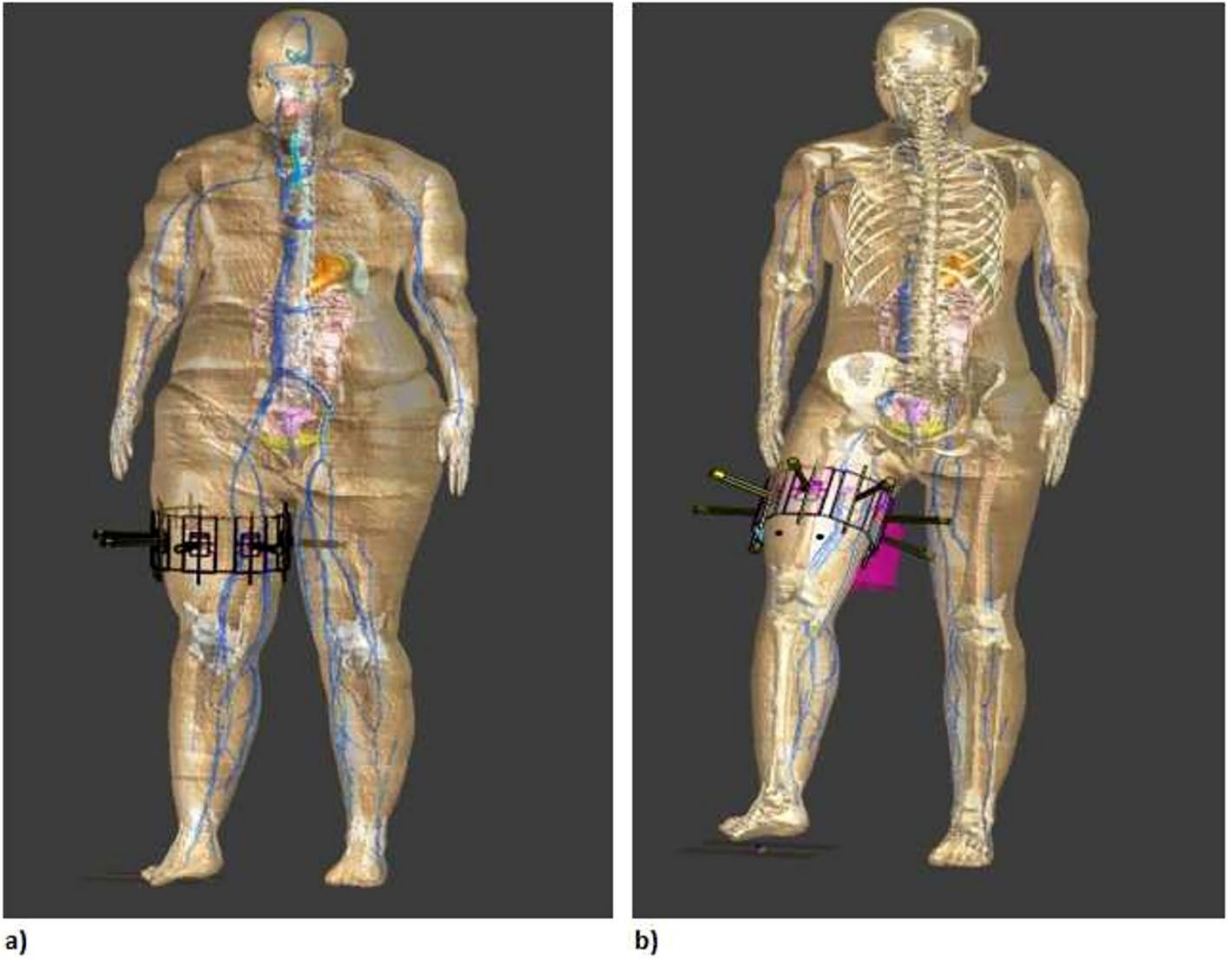
(**a**) Exemplar depiction of 8 antennas placed around Ella-30 model in static (standing pose). (**b**) Exemplar depiction of 8 antennas placed around Ella-22 model in a pose mimicking a phase of the gait cycle.

The following investigations were carried out using the four models:

- The antennas were placed around the thigh of the Duke model to image the femur. The model was in a static (standing) pose
- The antennas were placed around the thigh for each of the Ella models - Ella-22 (Ella with a BMI of 22), Ella-26 (Ella with a BMI of 26) and Ella-30 (Ella with a BMI of 30) - to image the femur. The models were in a static pose (Figure 3 a)
- The antennas placed around the thigh for the Ella-22 and Ella-30 models were rotated and translated with the thigh to image the femur in a pose mimicking a phase of the gait cycle (the hip flexed by $40^{\circ }$ followed by hip externally rotated by $5^{\circ }$ and with the knee flexed by $30^{\circ }$, Figure 3 b). The two models (Ella-22 and Ella-30) were chosen since they represent humans with a healthy and obese BMI score respectively.

**Table 1:** Characteristics of the four anatomical models used in this study. Duke is a male anatomical model with Ella-22 a female anatomical model of body mass index (BMI) score of 22. Ella-26 and Ella-30 are morphed models of Ella-22 with BMI scores of 26 and 30 respectively.

Anatomical model name	Age (years)	Sex	Height (m)	Mass (kg)	BMI ($\text {kg}/\text {m}^{2}$)
Duke	34	Male	1.74	70	23.1
Ella - 22	26	Female	1.63	57.3	22
Ella - 26	26	Female	1.63	69.4	26
Ella - 30	26	Female	1.63	79.7	30

In all the above cases, eight triangular patch monopole antennas were placed at equidistant points around the thigh. To ensure proper placement, a cylinder—whose circumference matched that of the thigh to be imaged—was used to calculate the antenna locations. The cylinder was attributed with electrical properties of the skin, thereby increasing the skin thickness in locations where the circumference of the cylinder was larger than that of the thigh. This ensured that the antennas were making direct contact with the skin.

Antenna locations in the posed model were determined based on a transformation matrix which was calculated using the location of three points on the skin during the standing pose and the location of the same three points in the posed model. This was to ensure that the deformations of the soft tissues and skin were accounted for when the model is posed and the antennas were shifted and that the antennas were not shifted purely due to the movement of the underlying bone. The deformations of the soft tissues and skin—due to the model transitioning from the standing pose to a pose mimicking a phase of the gait cycle (hip flexion of $40^{\circ }$, hip rotation of $5^{\circ }$ and knee flexion of $35^{\circ }$)—were calculated based on a physics simulation-based approach, wherein the user-prescribed motions of the bone are used to perform a tissue mechanical simulation of the deformations. These deformations also depend on the volume of soft tissues^55^, resulting in potentially different deformations between Ella-22 and Ella-30 models. The above steps were undertaken to ensure that the movement of antennas was similar to the movement wearable antennas would undergo in similar situations i.e the antennas would move based on soft tissue movement and not just the movement of bones.

#### Qualitative algorithms investigated

Four qualitative imaging algorithms were investigated: the confocal imaging algorithm, Delay and sum (DAS)^26^and its variant delay multiply and sum (DMAS)^56^; the multiple signal classification algorithm (MUSIC)^37,57^; and the Kirchhoff migration algorithm^58,59^. Implementations of DAS and DMAS found in the Microwave Radar-based Imaging Toolbox (MERIT)^60^were leveraged. Modifications to determine the imaging domain and the antenna delays were made to the functions to tailor them for our purposes. Additionally, an offset - determined using the breast tumour datasets provided in the toolbox - was subtracted from the true bone locations obtained from Sim4Life^53^ whilst investigating the validity of the reconstructed images.

MUSIC is based on the principle of time-reversal and has been extensively applied in microwave imaging applications. In our study, the multi-frequency variant of MUSIC was leveraged, where images reconstructed at each frequency were non-coherently summed to produce the final image. This was done to reduce artefacts in the reconstructed image and build on additional information obtained at multiple frequencies^37^. In addition, electric field values inside the thigh (computed in the simulations) were used in-lieu of the Green’s function as computation of Green’s functions for an inhomogenous background (the thigh) would be computationally expensive and inaccurate. Similarly, we have investigated a multi-frequency variant of Kirchhoff migration in this study. Kirchhoff migration has also been widely applied in microwave imaging applications^58,59^and has been reported to be a fast, stable and effective imaging technique for detecting small scatterers^61^. The multi-frequency variation of Kirchhoff migration was reported to produce better results than its single-frequency variations^61^.

The above four qualitative algorithms were investigated due to their widespread adoption in microwave imaging, their ability to generate images in real-time, and as their collected S-parameters can be directly applied to the imaging algorithms without any need for conversion to electric-field values. Additionally, only transmission parameters (S21 parameters) were provided to the imaging algorithms. This was decided based on literature evidence of transmission parameters collected from antennas diametrically opposite to the transmitting antenna^62,63^and from our initial investigation into microwave imaging^64^.

#### Simulations

In total, six investigations were carried out, one investigation for each of the models: Duke-Femur, Ella-22-Femur, Ella-26-Femur, Ella-30-Femur, Ella-22-Femur-Posed and Ella-30-Femur-Posed. For the first four investigations (performed on Duke-Femur, Ella-22-Femur, Ella-26-Femur and Ella-30-Femur), the following two simulations were performed:

- Simulation 1: where the bone (cortical and cancellous femur) was attributed with electrical properties of the muscle
- Simulation 2: where the bone (cortical and cancellous femur) was attributed with electrical properties of the bone

All scattering parameters (S-parameters) were collected through finite difference time domain (FDTD) simulations on Sim4Life. The results of Simulation 1 were used as the reference scan or empty scan data, which was then subtracted from the data of the second simulation prior to being used as input into the imaging algorithms. In addition, electric field values calculated using the first simulation were recorded at every pixel location inside the thigh to be used in-lieu of the Green’s function for the MUSIC and Kirchhoff migration algorithms. For femur detection in the posed models, empty scan data of Ella-22-Femur and Ella-30-Femur (Simulation 1) were subtracted from S-parameters acquired from Simulation 2 of the posed models.

For all simulations, a Gaussian pulse centered at the resonant frequency for each antenna and with a bandwidth of 3 GHz was used as the input waveform. The models were voxeled using the automatic voxelling tool in Sim4Life^53^.

#### Metrics

Visual verification of the reconstructed images was initially done to determine if a hotspot (indication of a scatterer) was present close to the true bone locations determined using Sim4Life^53^. In addition, metrics commonly applied in breast tumour detection were used to evaluate the accuracy of the reconstructed image^24,65^: Signal-to-cluster ratio (SCR), signal-to-mean ratio (SMR) and localisation error. SCR compares the maximum response inside the object of interest to the maximum response in the region outside the object of interest, and SMR compares the maximum response in the object of interest to the average response outside the object of interest. Localisation error is the euclidean distance between the expected centre of the object of interest (femur) to that of the maximum response in the image.

A higher SCR and SMR indicates a high-contrast localised region within the image. For example SCR and SMR values greater than 0 dB were obtained for breast tumour studies^65^, however negative SCR values were obtained for breast tumour detection in heterogeneous breasts. For all our investigations the object of interest was the bone (specifically, the femur).

## Results

### Performance of marker projection schemes

**Fig. 4:**
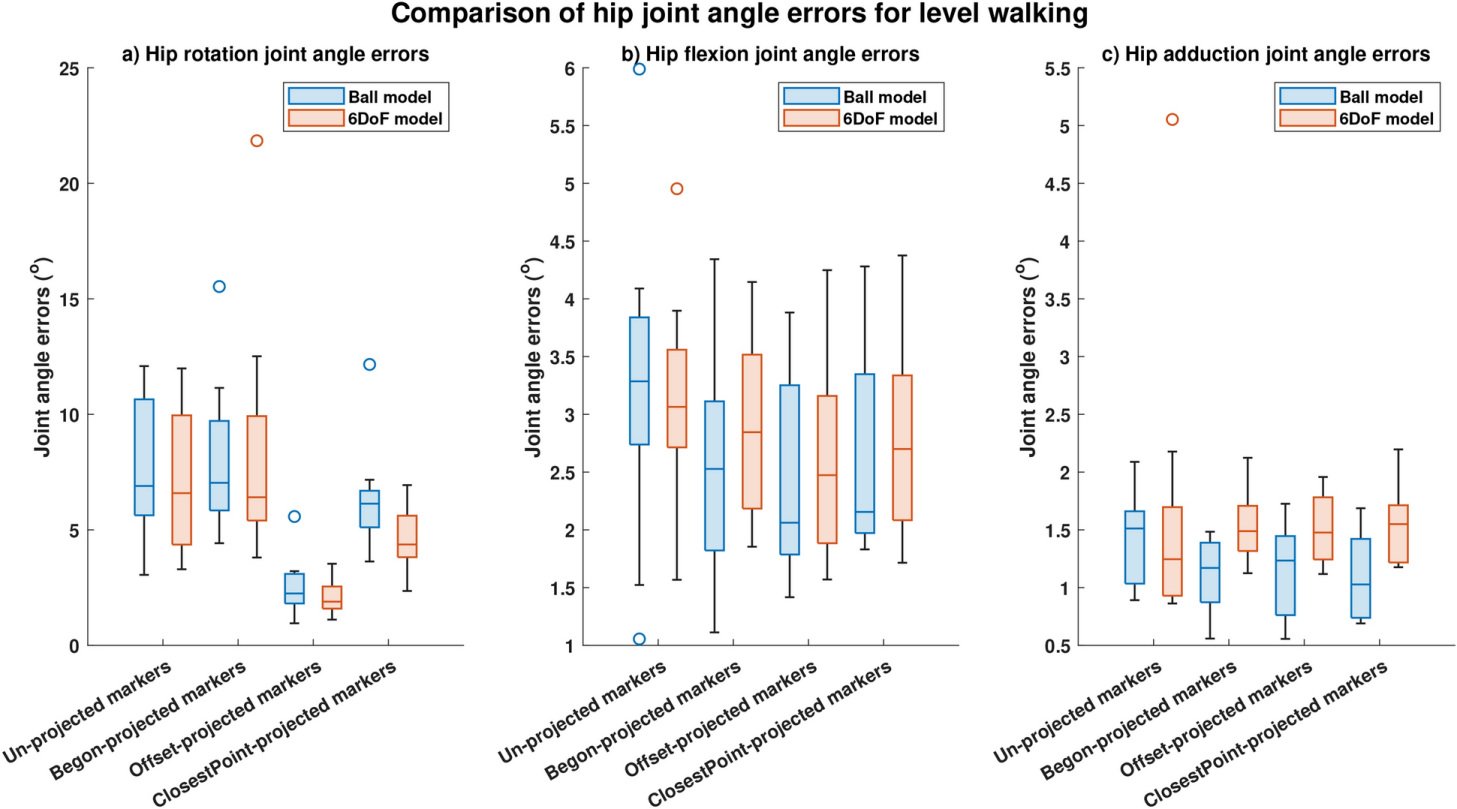
Joint angle errors computed during level walking. Comparison of median and spread of joint angle errors computed using four different projection methods (conventional (un-projected) skin-mounted marker, Begon-projection, offset-projection and closestPoint-projection) during level walking: (**a**) Hip rotation joint angle errors. (**b**) Hip flexion joint angle errors. (**c**) Hip adduction joint angle errors.

Substantial reductions in joint angle errors were obtained for both the models (Ball and 6DoF) using offset-projected and closestPoint-projected markers during all investigated motions (Figure 4-Figure 7). The performance of each projection scheme (determined by the degree of joint angle error) was compared with the performance of conventional (un-projected) skin-mounted markers for each studied motion.

Hip rotation errors computed using offset-projected markers significantly (p<0.05, Figure 4a) reduced during level walking for both the Ball model (reduction in error by 67.3%) and the 6DoF model (reduction in error by 78.8%). Considerable but non-significant reductions in hip rotation joint angle errors were obtained for level walking using closestPoint-projected markers and the Ball model (reduction in error by 11%) with significant (p<0.05) reduction in hip rotation joint angle errors obtained when the 6DoF model was leveraged (reduction in error by 33.7%, Figure 4a). Hip rotation errors obtained using Begon-projected markers increased by 1.9% for the Ball model and reduced by 2% for the 6DoF model with neither of the changes significant (Figure 4a).

In addition to reduction in hip rotation joint angle errors, the correlation (the degree of similarity) between hip rotation angles computed using offset-projected skin-mounted markers and those obtained using artefact-free bone movement increased from 0.59 (conventional (un-projected) skin-mounted markers) to 0.91 (Table 2) during level walking for the Ball model and from 0.60 (conventional (un-projected) skin-mounted markers) to 0.89 (offset-projected markers, Table 2) for the 6DoF model. However, the correlation values reduced for hip rotation angles computed using closestPoint-projected markers and Begon-projected markers (Table 2).

Whilst the projection of markers resulted in lower hip flexion and hip adduction joint angle errors when compared with conventional (un-projected) skin-mounted markers, none of the reductions were statistically significant (Figure 4b,c). The RMS joint angle errors and R2 values for level walking is given in Tables 2.

**Table 2:** Joint angle root mean square errors (RMSE) and correlation coefficients ($R^2$) for hip flexion, rotation and adduction angles computed using the different projection methods for level walking. Significance was computed as a comparison between joint angle errors obtained conventional (un-projected) markers and joint angle errors obtained using various marker projection schemes. RMSE, Root-mean-square-error;$R^2$, Correlation index.

Model	Joint Angles	Hip Flexion		Hip Rotation		Hip Adduction	
	Projections	RMSE ($^{\circ }$)	$R^2$	RMSE ($^{\circ }$)	$R^2$	RMSE ($^{\circ }$)	$R^2$
Ball joint	Skin-mounted markers (No projections)	3.2	0.994	7	0.592	1.4	0.967
	Begon Projection	2.5	0.995	8	0.391	1.1	0.975
	Offset Projection	2.4	0.995	**2.5** (p=5.854e-5)*	0.912	1.1	0.977
	Closest Point Projection	2.7	0.993	6.2	0.46	1.1	0.976
6 degree of freedom joint	Skin-mounted markers (No projections)	3.1	0.991	7	0.605	1.6	0.973
	Begon Projection	2.8	0.989	8.5	0.217	1.5	0.957
	Offset Projection	2.6	0.993	**2.0** (p=5.979e-5)*	0.889	1.5	0.966
	Closest Point Projection	2.8	0.992	**4.6** (p=0.0309)*	0.247	1.5	0.954

Bold and * : p < 0.05.

**Fig. 5:**
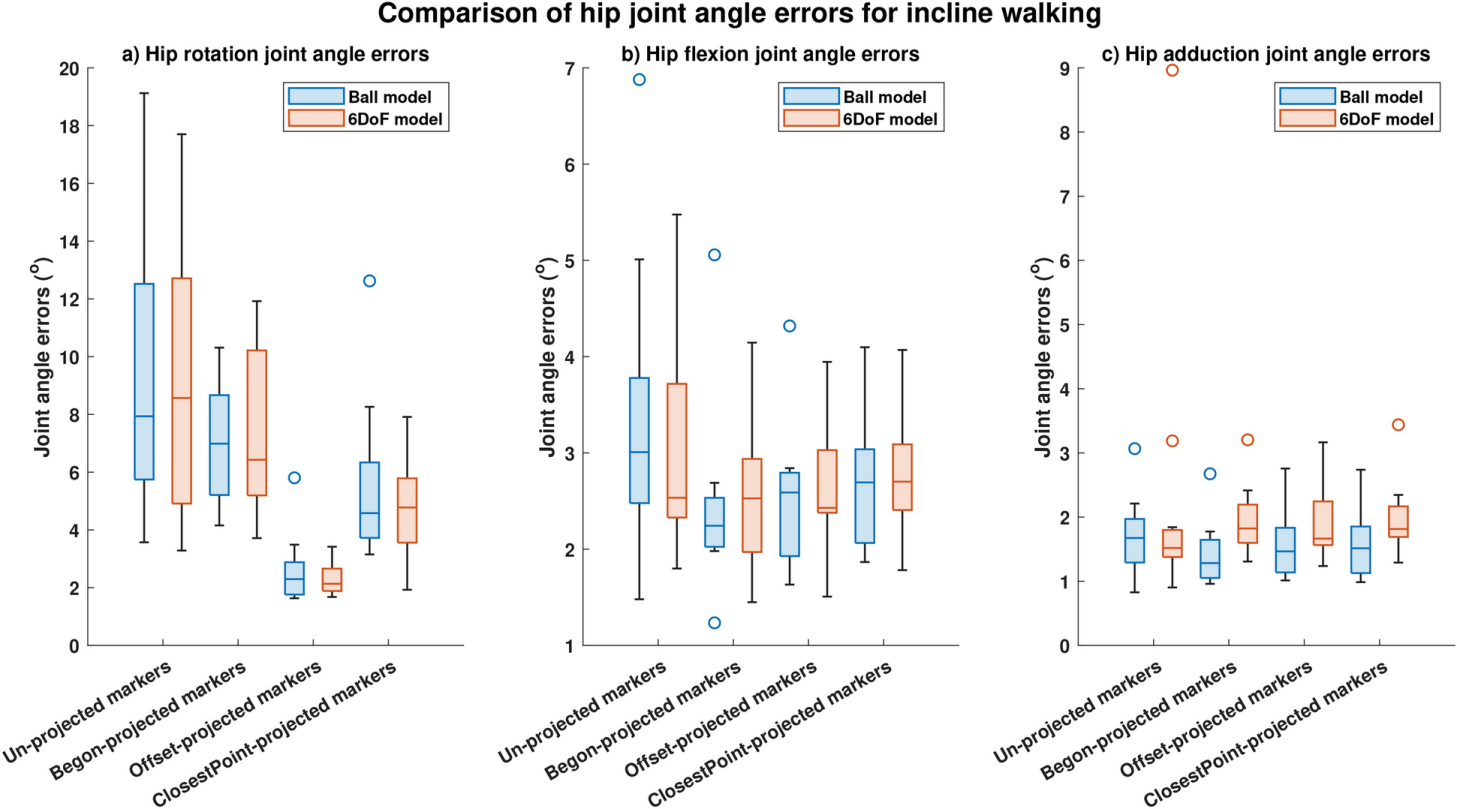
Joint angle errors computed during incline walking. Comparison of median and spread of joint angle errors computed using four different projection methods (conventional (un-projected) skin-mounted marker, Begon-projection, offset-projection and closestPoint-projection) during incline walking: (**a**) Hip rotation joint angle errors. (**b**) Hip flexion joint angle errors. (**c**) Hip adduction joint angle errors.

Similar results to those obtained during level walking were also obtained during incline walking with significant (p<0.05) reductions in hip joint angle errors obtained using offset-projected markers and closest-point projected markers for both the models (Ball model: reductions of 71.0% using offset-projected markers and of 42.2% using closestPoint-projected markers; 6DoF model: reductions of 75.1% for offset-projected markers and reductions of 44.2% for closestPoint-projected markers) (Figure 5 a). Hip rotation errors obtained using Begon-projected markers reduced by 11.9% for the Ball model and significantly (p<0.05) by 24.9% for the 6DoF model (Figure 5 a). Reductions in hip flexion joint angle errors and hip adduction joint angle errors were not significant (Figure 5 b,c).

Correlation computed using offset-projected markers increased from 0.7094 (conventional (un-projected) skin-mounted markers) to 0.82 (offset-projected markers during incline walking for the Ball model and from 0.71 (conventional (un-projected) skin-mounted markers) to 0.83 for the 6DoF model. Correlation values computed using closestPoint-projected markers and Begon-projected markers were similar to or lesser than those obtained using conventional (un-projected) skin-mounted markers (Table 3).

**Table 3:** Joint angle root mean square errors (RMSE) and correlation coefficients ($R^2$) for hip flexion, rotation and adduction angles computed using the different projection methods for incline walking. Significance was computed as a comparison between joint angle errors obtained conventional (un-projected) markers and joint angle errors obtained using various marker projection schemes. RMSE, Root-mean-square-error;$R^2$, Correlation index.

Model	Joint Angles	Hip Flexion		Hip Rotation		Hip Adduction	
	Projections	RMSE ($^{\circ }$)	$R^2$	RMSE ($^{\circ }$)	$R^2$	RMSE ($^{\circ }$)	$R^2$
Ball joint	Skin-mounted markers (No projections)	3.4	0.996	9.2	0.709	1.7	0.96
	Begon Projection	2.4	0.997	**7.0 ** (p=1.32e-6)*	0.521	**1.4 ** (p=0.04)*	0.97
	Offset Projection	2.5	0.996	**2.7** (p=1.48e-6)*	0.823	1.6	0.967
	Closest Point Projection	2.7	0.996	**5.6** (p=2.58e-5)*	0.528	1.6	0.966
6 degree of freedom joint	Skin-mounted markers (No projections)	2.9	0.995	8.9	0.706	2.3	0.969
	Begon Projection	2.5	0.995	**7.4 ** (p=5.2e-5)*	0.406	1.9	0.963
	Offset Projection	**2.7 ** (p=0.01)*	0.996	**2.3 ** (p=4.54e-7)*	0.832	1.9	0.962
	Closest Point Projection	**2.8 ** (p=0.01)*	0.995	**4.8 ** (p=5.56e-7)*	0.35	1.9	0.959

Bold and * : p < 0.05.

**Fig. 6:**
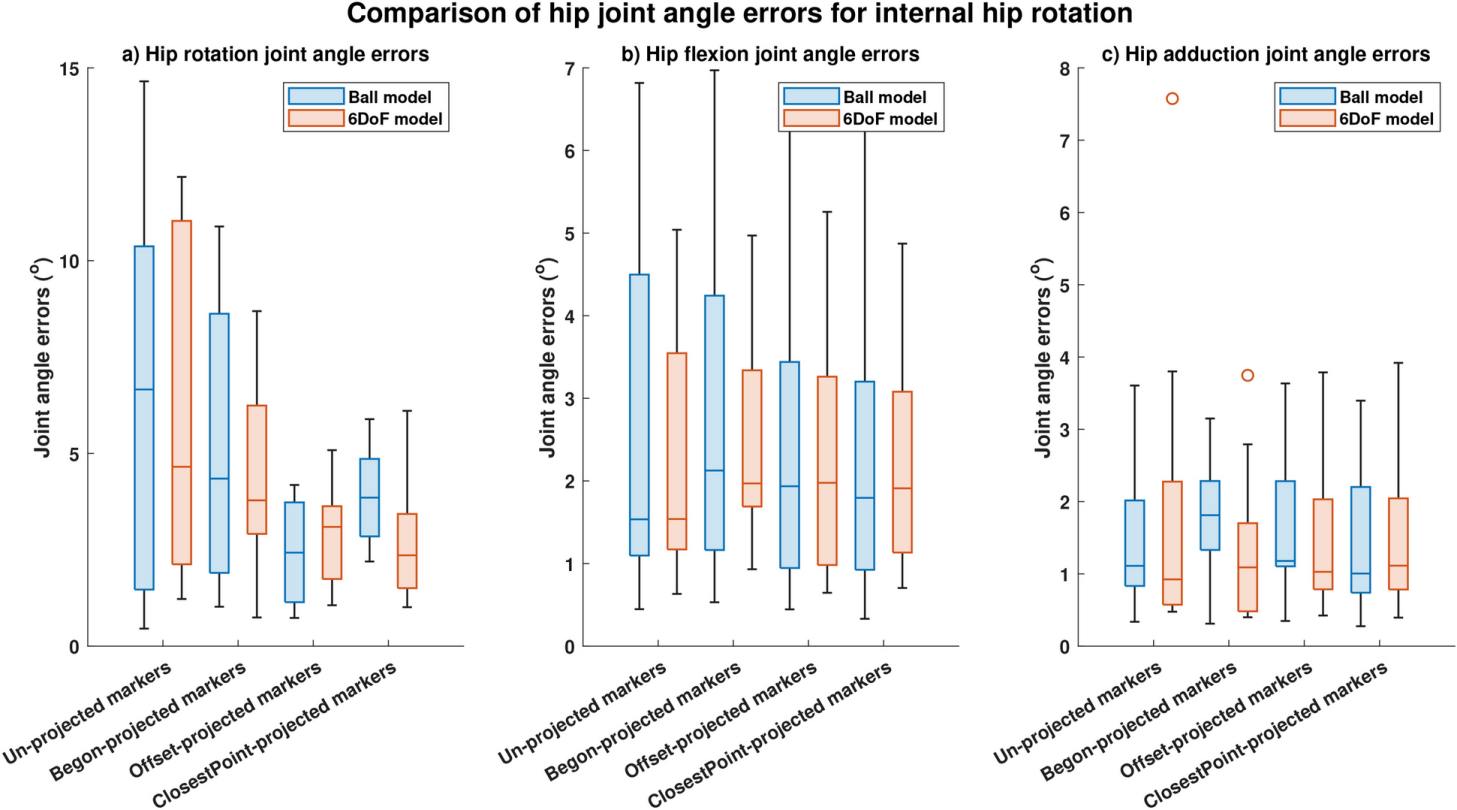
Joint angle errors computed during internal rotation. Comparison of median and spread of joint angle errors computed using four different projection methods (conventional (un-projected) skin-mounted marker, Begon-projection, offset-projection and closestPoint-projection) during internal hip rotation: (**a**) Hip rotation joint angle errors. (**b**) Hip flexion joint angle errors. (**c**) Hip adduction joint angle errors.

**Fig. 7:**
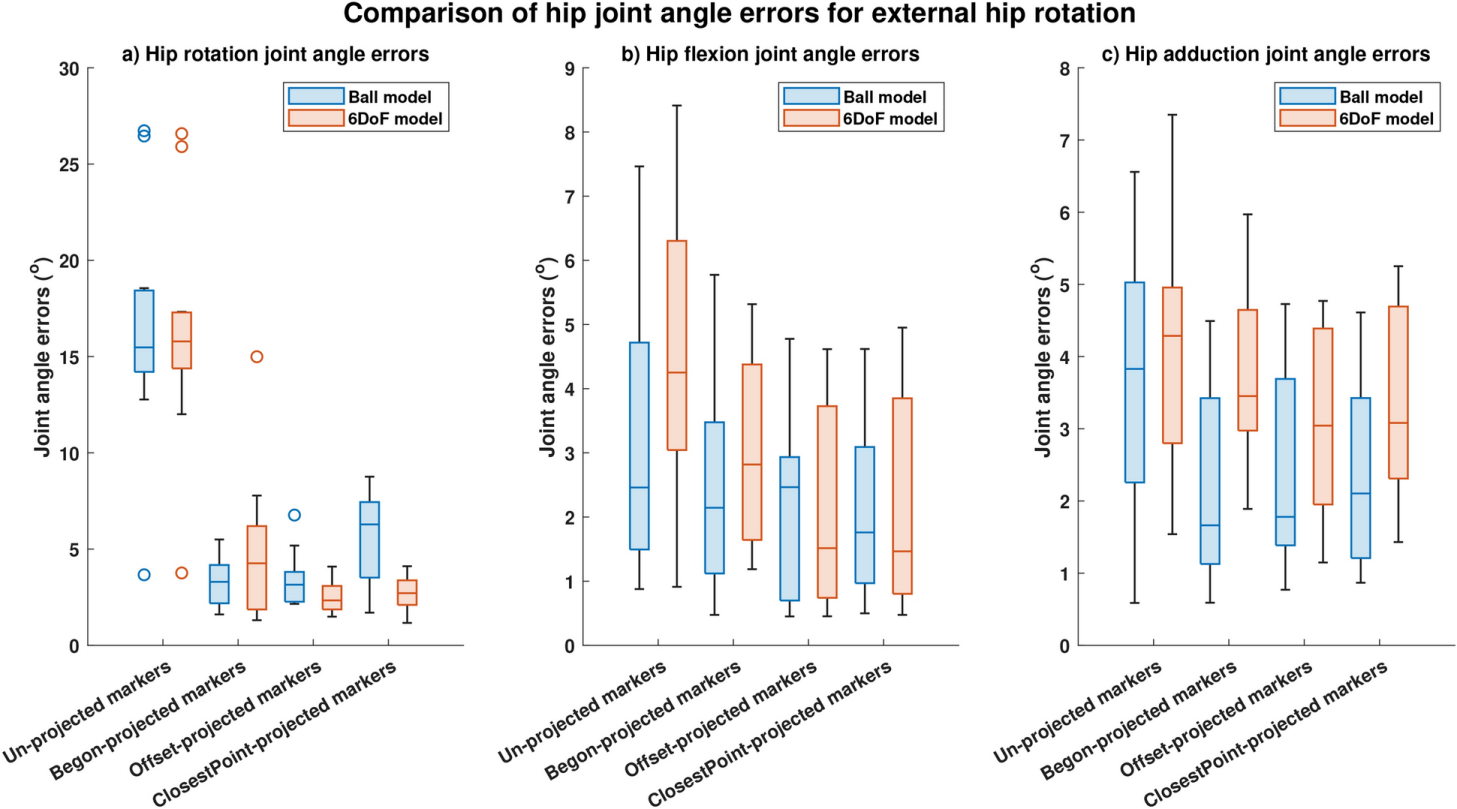
Joint angle errors computed during external rotation. Comparison of median and spread of joint angle errors computed using four different projection methods (conventional (un-projected) skin-mounted marker, Begon-projection, offset-projection and closestPoint-projection) during external hip rotation: (**a**) Hip rotation joint angle errors. (**b**) Hip flexion joint angle errors. (**c**) Hip adduction joint angle errors.

For internal and external hip rotation motions, hip rotation joint angle errors computed using offset-projected markers significantly (p<0.05) reduced by 63.5% and by 79.6% respectively for the Ball model (Figures 6a, 7a, p < 0.05) and by 33.5% and 85.2% respectively for the 6DoF model (Figures 6a, 7a, p < 0.05). Non-significant reductions in hip rotation joint angle errors were computed using closestPoint-projected markers for internal hip rotation (Ball model: reductions of 59.4%; 6DoF model: reductions by 82.8%). Significant reductions (p<0.05) in hip rotation angle errors during external hip rotation were obtained using Begon-projected markers with reductions of 78.6% obtained for the Ball model and reductions of 73.0% for the 6DoF model (Figure 7a). Non-significant reductions of 34.7% (Ball model) and 18.7% (6DoF model) were obtained using Begon-projected markers during internal hip rotation.

Additionally, correlation with artefact-free bone movement increased for hip rotations computed using offset-projected markers and both the Ball model (0.80->0.89) and the 6DoF model (0.78->0.88) during external hip rotation (Table 5). For internal hip rotation, correlation of hip rotation angles computed using offset-projected markers increased for the 6DoF model (0.80->0.81) but decreased for the Ball model (0.90->0.87); no improvements were observed when using closestPoint-projected markers or Begon-projected markers (Table 4).

**Table 4:** Joint angle root mean square errors (RMSE) and correlation coefficients ($R^2$) for hip flexion, rotation and adduction angles computed using the different projection methods for internal hip rotation. Significance was computed as a comparison between joint angle errors obtained conventional (un-projected) markers and joint angle errors obtained using various marker projection schemes. RMSE, Root-mean-square-error;$R^2$, Correlation index.

Model	Joint Angles	Hip Flexion		Hip Rotation		Hip Adduction	
	Projections	RMSE ($^{\circ }$)	$R^2$	RMSE ($^{\circ }$)	$R^2$	RMSE ($^{\circ }$)	$R^2$
Ball joint	Skin-mounted markers (No projections)	2.7	0.971	6.2	0.902	1.4	0.964
	Begon Projection	2.7	0.954	5.2	0.666	1.8	0.965
	Offset Projection	2.4	0.969	**2.4** (p=0.03)*	0.87	1.6	0.968
	Closest Point Projection	2.3	0.971	3.9	0.605	1.5	0.967
6 degree of freedom joint	Skin-mounted markers (No projections)	2.3	0.967	6.5	0.809	1.8	0.937
	Begon Projection	2.5	0.905	4.3	0.657	1.4	0.946
	Offset Projection	2.3	0.967	**2.9** (p=4.5e-7)*	0.818	1.5	0.947
	Closest Point Projection	2.2	0.964	**2.8** (p=0.022)*	0.422	1.6	0.945

Bold and * : p < 0.05.

**Table 5:** Joint angle root mean square errors (RMSE) and correlation coefficients ($R^2$) for hip flexion, rotation and adduction angles computed using the different projection methods for external hip rotation. Significance was computed as a comparison between joint angle errors obtained conventional (un-projected) markers and joint angle errors obtained using various marker projection schemes. RMSE, Root-mean-square-error;$R^2$, Correlation index.

Model	Joint Angles	Hip Flexion		Hip Rotation		Hip Adduction	
	Projections	RMSE ($^{\circ }$)	$R^2$	RMSE ($^{\circ }$)	$R^2$	RMSE ($^{\circ }$)	$R^2$
Ball joint	Skin-mounted markers (No projections)	3.3	0.709	16.6	0.809	3.7	0.706
	Begon Projection	2.5	0.693	**3.3** (p=1.32e-6)*	0.663	**2.2** (p=0.04)*	0.772
	Offset Projection	2.1	0.767	**3.4** (p=1.48e-6)*	0.893	2.3	0.779
	Closest Point Projection	2.1	0.629	**5.6** (p=2.58e-5)*	0.649	2.3	0.757
6 degree of freedom joint	Skin-mounted markers (No projections)	4.4	0.46	16.3	0.782	4.1	0.682
	Begon Projection	2.9	0.117	**4.9** (p=5.2e-5)*	0.649	3.7	0.614
	Offset Projection	**2.1** (p=0.01)*	0.591	**2.5** (p=4.54e-7)*	0.887	3.1	0.731
	Closest Point Projection	**2.2** (p=0.01)*	0.477	**2.7** (p=5.56e-7)*	0.542	3.4	0.64

Bold and * : p<0.05.

Reductions in hip flexion angle errors and hip adduction angle errors were not significant for internal and external hip rotations using any marker projection schemes. Of note, hip flexion joint angle errors computed using conventional (un-projected) skin-mounted markers and 6DoF model was lower for the internal hip rotation motion when compared with offset-projected markers and Begon-projected markers (Figure 7). Hip adduction joint angle errors computed using conventional (un-projected) skin-mounted markers and the Ball model was lower when compared with offset-projected markers and Begon-projected markers (Figure 6). The RMS joint angle errors and correlation (R2) values for internal and external hip rotations are given in Tables 4- 5.

**Fig. 8:**
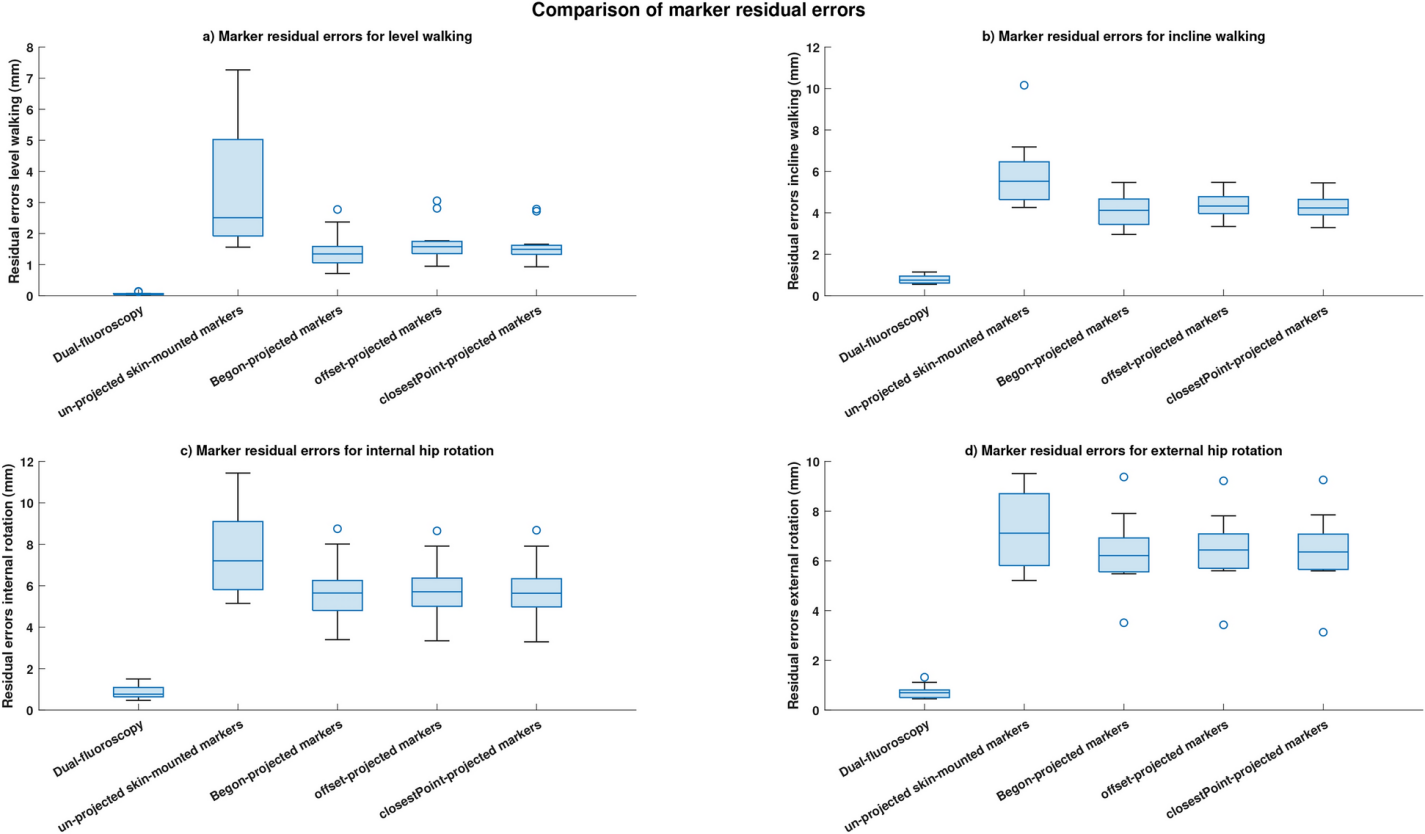
Comparisons of residual errors between the five markersets. Comparison of residual errors between five markersets obtained for all investigated motions. (**a**) Residual errors obtained during level walking. (**b**) Residual errors obtained during incline walking. (**c**) Residual errors obtained during internal hip rotation. (**d**) Residual errors obtained during external hip rotation.

Residual errors computed using projected markers were lesser than those computed using conventional (un-projected) skin-mounted markers for all investigated motions and for both the Ball and 6DoF model(Figure 8).

### Viability of applying microwave imaging in biomechanical applications

Our results indicate that the location of the bone from the skin surface was determined for every anatomical model using microwave imaging. Additionally, the location of the bone was determined both in the static pose and in a pose mimicking a phase of the gait cycle.

The images were reconstructed using four qualitative imaging algorithms: DAS, DMAS, MUSIC and Kirchhoff migration. As detailed in the methods section, the MERIT toolbox was leveraged to reconstruct images using DAS and DMAS with an offset correction - calculated using the breast tumour dataset available in the MERIT toolbox - applied to the true bone locations calculated in Sim4Life^53^.

**Table 6:** Metrics of reconstructed images computed using data collected from antenna 3. Values with ’-’ indicate the bone could not be determined in the reconstructed image. SCR, signal-to-cluster ratio; SMR, signal-to-mean ratio; DAS, delay-and-sum confocal imaging; DMAS, delay-multiply-and-sum confocal imaging; MUSIC, multiple signal classification.

Anatomical model	Algorithm	SMR (dB)	SCR (dB)	Localisation error (cm)
Duke	DAS	18.8088	−0.3083	1.9008
	DMAS	26.8214	−0.6868	1.7946
	MUSIC	5.114	−2.557	1.4052
	Kirchhoff	6.0287	−3.3893	0.6969
Ella - 22	DAS	13.98	−2.7514	1.1133
	DMAS	14.57	−7.5473	1.1752
	MUSIC	5.3665	−2.7663	1.48
	Kirchhoff	2.0461	−3.241	0.9815
Ella - 26	DAS	18.8732	−3.0647	1.0533
	DMAS	23.3552	−6.8105	1.0533
	MUSIC	8.245	−1.9048	1.75
	Kirchhoff	6.7864	−1.3686	1.9332
Ella - 30	DAS	19.4456	−3.7721	0.7647
	DMAS	24.641	−6.179	0.7647
	MUSIC	7.1731	−1.3928	2.85
	Kirchhoff	5.145	−1.2754	2.488
Ella - 22 Posed	DAS	-	-	-
	DMAS	-	-	-
	MUSIC	2.8905	−4.913	2.6766
	Kirchhoff	−0.1612	−4.7139	2.0088
Ella - 30 Posed	DAS	-	-	-
	DMAS	-	-	-
	MUSIC	7.8944	−3.3131	1.6901
	Kirchhoff	−1.2796	−3.67141	1.202

**Fig. 9:**
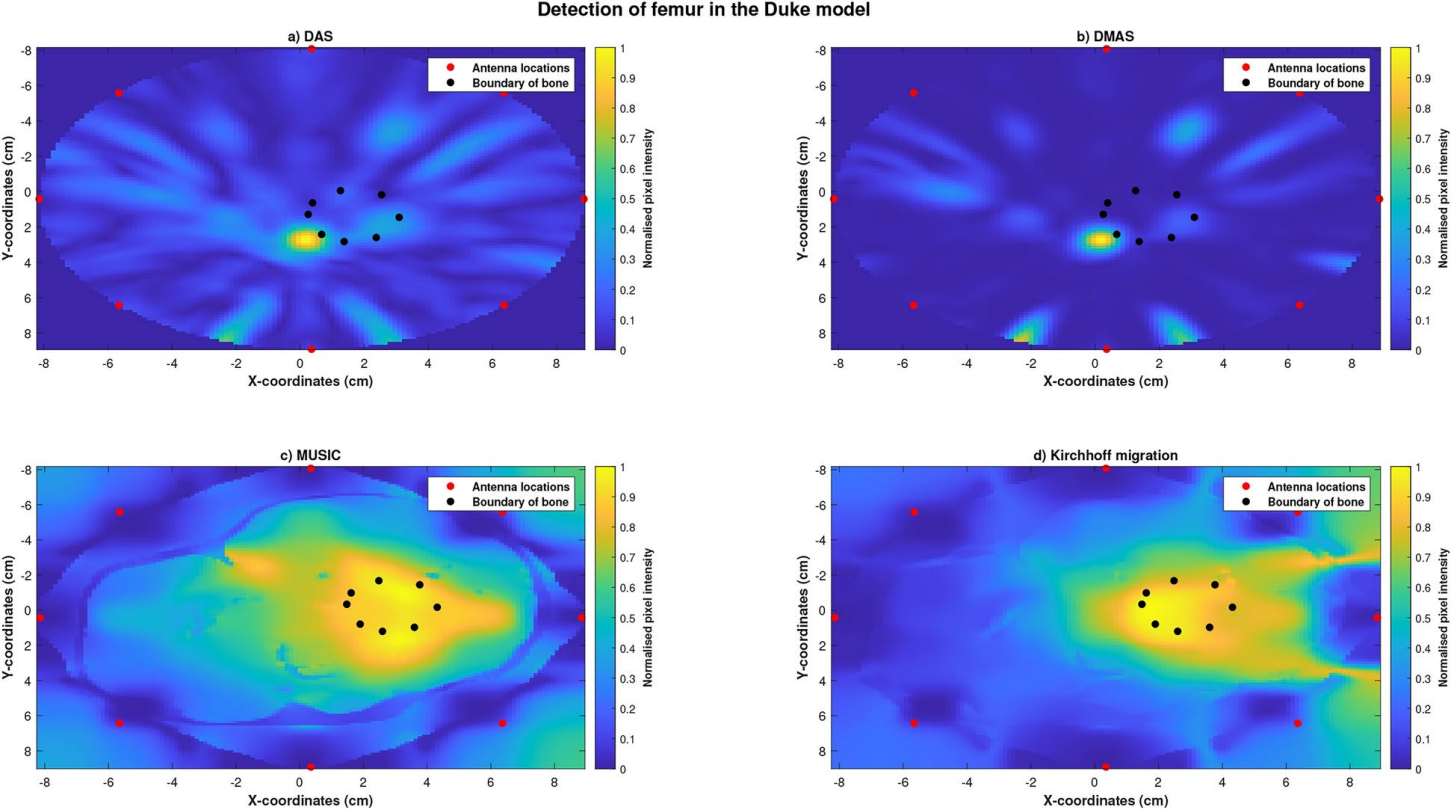
Reconstructed images of the thigh and femur using antenna 3 and the duke anatomical model. The hotspot indicates the location of the bone. The black dots indicate the true circumference of the bone as obtained from Sim4Life and the red dots indicate the location of the antennas. (**a**) Reconstructed image obtained using delay-and-sum (DAS). (**b**) Reconstructed image obtained using delay-multiply-and-sum (DMAS). (**c**) Reconstructed image obtained using multiple signal classification (MUSIC). (**d**) Reconstructed image obtained using Kirchhoff migration. The colour scale represents the normalised magnitude of scattering calculated using the imaging algorithms.

The location of the femur in the Duke anatomical model was visually detected using each of the four imaging algorithms (Figure 9). SMR was highest for the image reconstructed using DMAS (SMR: 26.8214 dB) and lowest for the image reconstructed using MUSIC (SMR: 5.11 dB). Images reconstructed using DAS had the highest SCR (SCR: −0.3083 dB) with images reconstructed using Kirchhoff migration having the lowest (SCR: −3.3 dB). Localisation error was the lowest for images reconstructed using Kirchhoff migration (Localisation error: 0.69 cm), with maximum error obtained using the DAS algorithm (Localisation error:1.90 cm, Table 6).

**Fig. 10:**
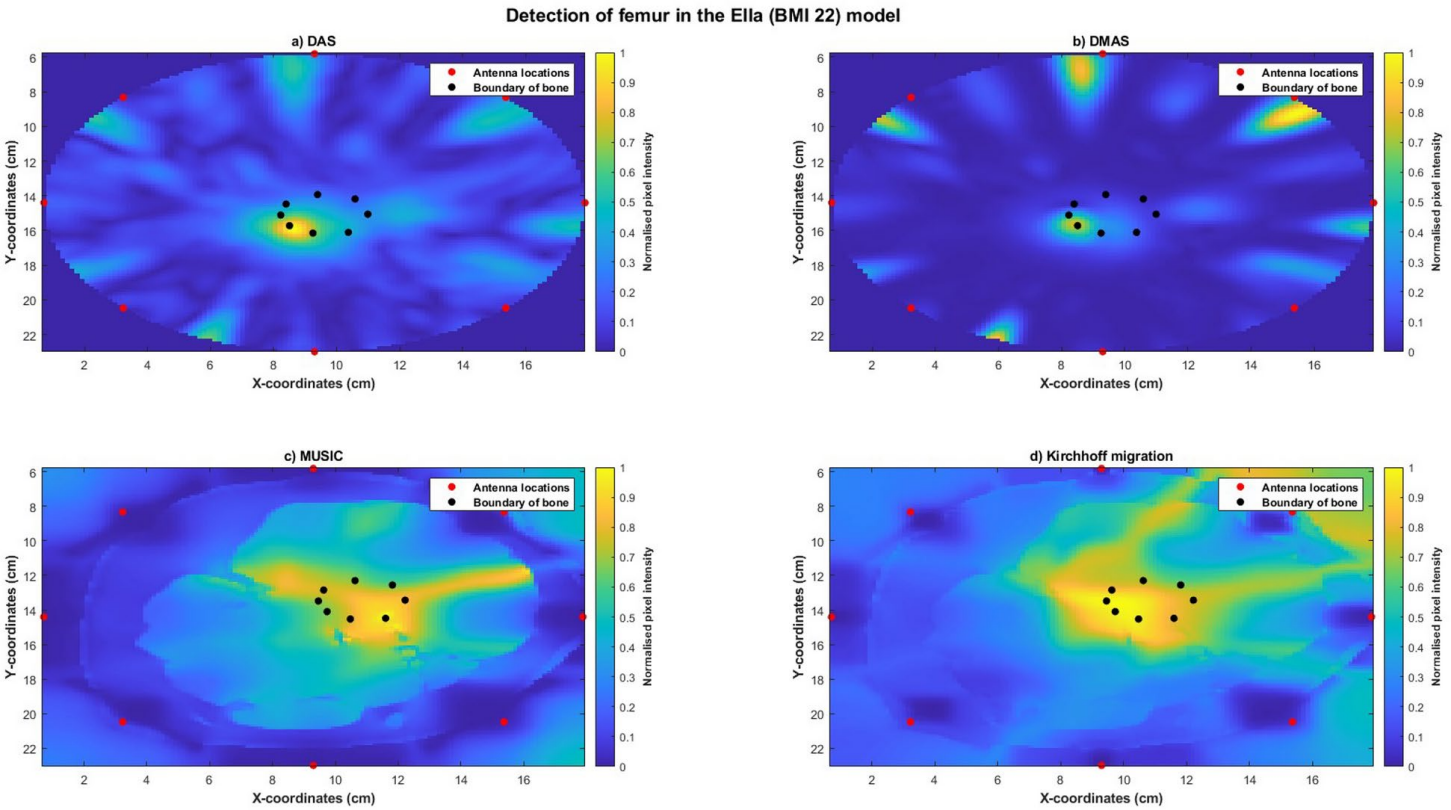
Reconstructed images of the thigh and femur using antenna 3 and the Ella-22 anatomical model. The hotspot indicates the location of the bone. The black dots indicate the true circumference of the bone as obtained from Sim4Life and the red dots indicate the location of the antennas. (**a**) Reconstructed image obtained using delay-and-sum (DAS). (**b**) Reconstructed image obtained using delay-multiply-and-sum (DMAS). (**c**) Reconstructed image obtained using multiple signal classification (MUSIC). (**d**) Reconstructed image obtained using Kirchhoff migration. The colour scale represents the normalised magnitude of scattering calculated using the imaging algorithms.

**Fig. 11:**
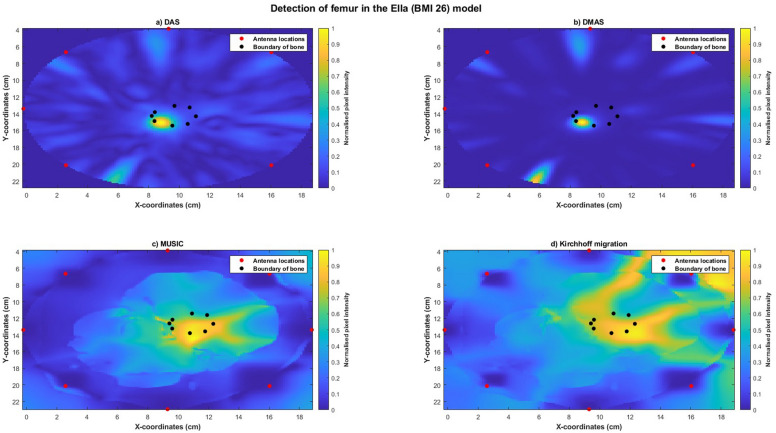
Reconstructed images of the thigh and femur using antenna 3 and the Ella-26 anatomical model. The hotspot indicates the location of the bone. The black dots indicate the true circumference of the bone as obtained from Sim4Life and the red dots indicate the location of the antennas. (**a**) Reconstructed image obtained using delay-and-sum (DAS). (**b**) Reconstructed image obtained using delay-multiply-and-sum (DMAS). (**c**) Reconstructed image obtained using multiple signal classification (MUSIC). (**d**) Reconstructed image obtained using Kirchhoff migration. The colour scale represents the normalised magnitude of scattering calculated using the imaging algorithms.

**Fig. 12:**
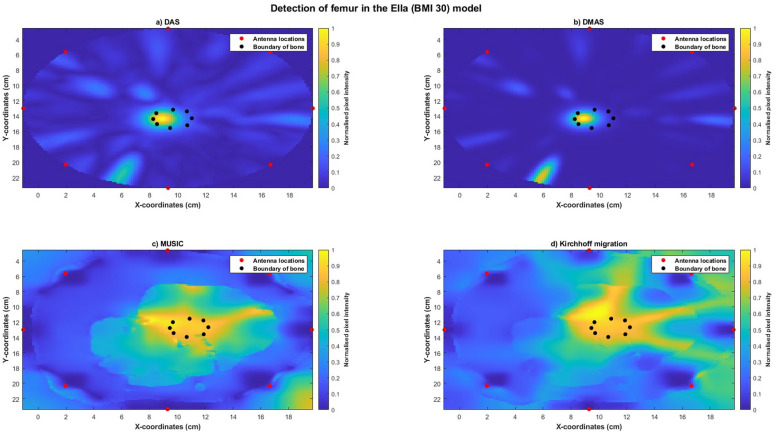
Reconstructed images of the thigh and femur using antenna 3 and the Ella-30 anatomical model. The hotspot indicates the location of the bone. The black dots indicate the true circumference of the bone as obtained from Sim4Life and the red dots indicate the location of the antennas. (**a**) Reconstructed image obtained using delay-and-sum (DAS). (**b**) Reconstructed image obtained using delay-multiply-and-sum (DMAS). (**c**) Reconstructed image obtained using multiple signal classification (MUSIC). (**d**) Reconstructed image obtained using Kirchhoff migration. The colour scale represents the normalised magnitude of scattering calculated using the imaging algorithms.

Reconstructed images of Ella-22 (Figure 10), Ella-26 (Figure 11) and Ella-30 (Figure 12) visually indicated the location of the bone when imaged using all four imaging algorithms. SMR and SCR values generally increased with BMI scores for each imaging algorithm with maximum SMR (DAS: 19.4456 dB, DMAS: 24.6410 dB, MUSIC: 7.1731 dB, Kirchhoff: 5.14 dB) and SCR (DAS: −3.7721 dB, DMAS: −6.1790 dB, MUSIC: −1.3928 dB, Kirchhoff: −1.27 dB) obtained for the Ella-30 model. Localisation errors for images reconstructed using confocal imaging (DAS and DMAS) reduced with higher BMI scores, with the smallest error obtained for the Ella-30 model (DMAS: 0.7647 mm). However, localisation errors obtained using MUSIC and Kirchhoff migration increased with BMI scores (Table 6). Within each model, SMR was highest for images reconstructed using DMAS with highest SCR obtained using Kirchhoff migration for Ella-26 and Ella-30 models. While comparable localisation errors were obtained between the four algorithms for Ella-22, localisation errors obtained using the confocal imaging algorithms were lesser than those obtained using MUSIC or Kirchhoff migration for Ella-26 and Ella-30 models.

**Fig. 13:**
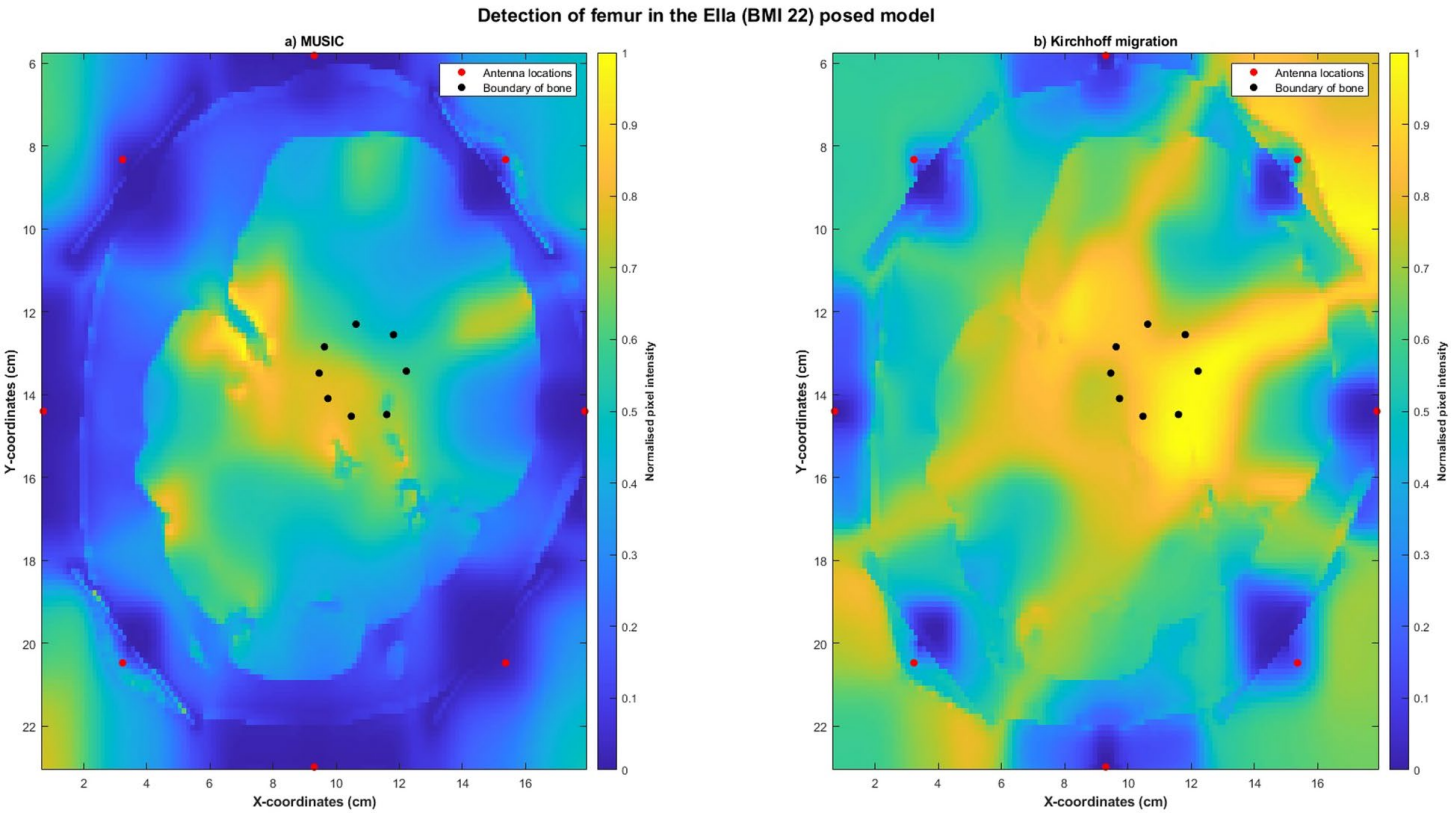
Reconstructed images of the thigh and femur using antenna 3 and the Ella-22 anatomical model in a pose mimicking a phase of the gait cycle. The hotspot indicates the location of the bone. The black dots indicate the true circumference of the bone as obtained from Sim4Life and the red dots indicate the location of the antennas. (**a**) Reconstructed image obtained using multiple signal classification (MUSIC). (**b**) Reconstructed image obtained using Kirchhoff migration. The colour scale represents the normalised magnitude of scattering calculated using the imaging algorithms.

**Fig. 14:**
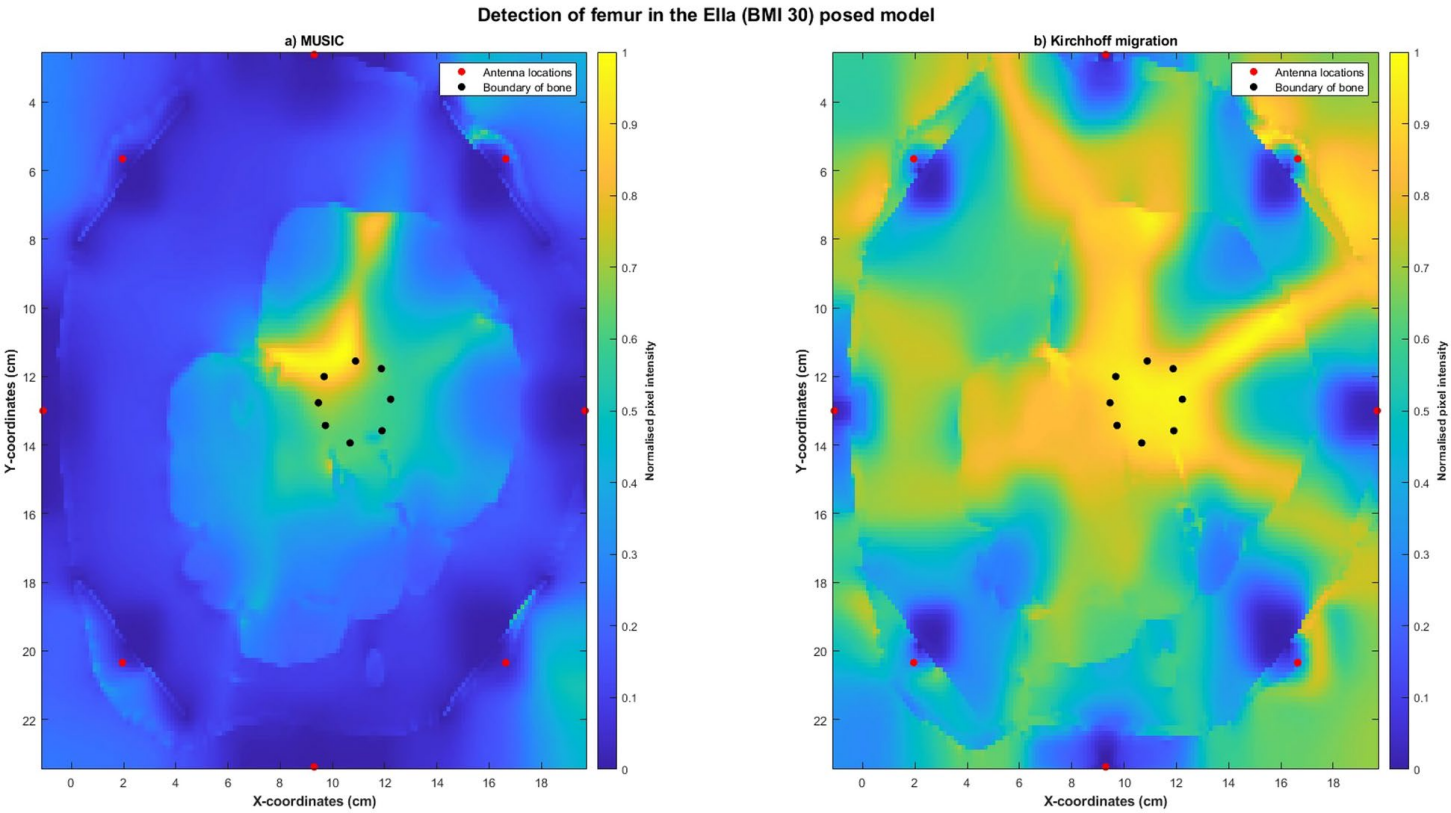
Reconstructed images of the thigh and femur using antenna 3 and the Ella-30 anatomical model in a pose mimicking a phase of the gait cycle. The hotspot indicates the location of the bone. The black dots indicate the true circumference of the bone as obtained from Sim4Life and the red dots indicate the location of the antennas. (**a**) Reconstructed image obtained using multiple signal classification (MUSIC). (**b**) Reconstructed image obtained using Kirchhoff migration. The colour scale represents the normalised magnitude of scattering calculated using the imaging algorithms.

Reconstructed images obtained using MUSIC and Kirchhoff migration methods for the Ella-22-posed (Figure 13) and the Ella-30-posed (Figure 14) models visually indicated the location of the bone. Localisation errors were within 2.5cm for both models using both the imaging algorithms, with SMR calculated using MUSIC greater than that calculated using Kirchhoff migration. Reconstructed images obtained using confocal imaging algorithms (DAS and DMAS) failed to produce images of the bone.

## Discussion

The results of our study indicate that: 1). The projection of skin-mounted markers onto the bone surface significantly reduces joint angle errors when compared with joint angle errors acquired using conventional methods of movement analysis (un-projected skin-mounted markers) and other marker-projection schemes, thereby improving kinematic accuracy (specifically in computing hip rotation angles in the coronal and transverse plane); and 2). The femur can be successfully detected from the skin surface - in both static and posed configurations - using microwave imaging with data collected from a limited number of wearable antennas in the absence of coupling liquid, thereby underscoring the viability of leveraging microwave imaging in biomechanical applications, specifically to locate the bone from the skin surface to enable marker projection.

### Projection of markers

Our results indicate that in addition to reducing joint angle errors, hip kinematics computed using our proposed marker-projection schemes had an increase in correlation with hip kinematics computed using artefact-free bone movement (true hip movement) when compared with the correlation obtained using conventional (un-projected) skin-mounted markers, indicating an increase in the quality of the computed kinematics. Both joint angle errors and correlation were computed against kinematics obtained from artefact-free bone movement acquired using dual-fluoroscopy which do not suffer from STA and can be considered as ground-truth kinematics, and thereby indicates the efficacy of our method in reducing the influence of STA on computed kinematics.

Previous studies quantifying STA and its impact on kinematics have indicated that internal/external rotation and abduction/adduction are significantly affected by STA, with the magnitude of joint angle errors comparable to that of actual bone movement^66–68^. In their study using the same dataset as the one used in this study^45,46,69^, the authors reported that hip joint angles computed using conventional (un-projected) skin-mounted markers were underestimated compared with kinematics computed using DF-markers, and that neither the DoF of the model (joint constraints) nor the marker configuration reduced joint angle errors during walking or hip rotation activities^45^. Additionally, they reported joint angle errors of 8 degrees, 9 degrees, 12 degrees and 8 degrees using conventional (un-projected) skin-mounted markers in rotation during level walking, incline walking, internal abduction and external abduction respectively^45,46^. Their findings, one of the first to report the effect of STA on hip kinematics, underscored the need for a solution to ameliorate the effects of STA.

Whilst we obtained similar hip rotation errors using conventional (un-projected) markers, we noted a significant (p<0.05) decrease in internal/external rotation errors during level walking, incline walking and internal and external hip rotations using markers projected on to the bone surface. Additionally, we observed lower hip flexion and adduction errors during all activities using projected markers compared with conventional (un-projected) markers; however, these differences were not significant. These results underscore the efficacy of our proposed methods to reduce the effects of STA on computed kinematics, specifically in rotations most affected by STA.

We also obtained lower kinematic errors, through projection of markers, for models incorporating different joint constraints. The efficacy of various methods proposed to reduce the effects of STA have been reported to vary based on the joint constraint incorporated^15^, with the 6DoF and ball joints reported to produce kinematics and kinetics with higher accuracy than anatomically realistic joint constraints^70,70–72^. We tested our projection schemes on models incorporating both 6DoF and ball joints, with our results indicating improvements in computed hip rotation, flexion and adduction angles for both the models, underscoring the generalisability and applicability of our proposed method.

Our results also indicate that improved joint angle estimation is obtained using our marker-projection schemes when compared with markers projected onto the longitudinal axis of the segment^15^. Whilst the projection of a subset of markers (or markers on the cuff) was reported to reduce joint angle errors in the upper extremity body during activities of daily living^15^, the projection of all markers onto the longitudinal axis increased joint angle error. The cause of this increase in joint angle error was attributed to a loss of 3d information, which occurs when the coordinates of two axes were made to 0^15^.

In our study, we observed that, whilst the projection of markers onto the longitudinal axis does improve kinematic accuracy, it is also associated with a reduction in correlation with the actual bone movement when compared with conventional (un-projected) skin-mounted markers. These issues were ameliorated by projecting markers onto the bone surface, with offset-projected markers improving the quality of joint angle estimation (as indicated by improved correlation compared with skin-mounted markers) and closestPoint-projected markers indicating a similar correlation to those obtained using markers projected onto the longitudinal axis.

Our results additionally indicate that projecting skin-mounted markers onto the bone surface (our marker-projection schemes) produces lesser residual errors. Residual errors are used as a goodness-of-fit metric between the underlying model and the experimental data^49,51,73^, with lower residual errors reported to be an indication of superior pose reconstruction capability of the proposed model^74^and improved STA compensation^75^. Therefore, our results indicate that projecting the markers onto the bone surface improves kinematic accuracy as indicated by lower joint angle errors.

The reductions in joint angle errors computed using our proposed marker projection schemes were greater than those obtained using various non-marker projection STA compensation methods reported in literature such as novel pose estimators^76^, novel joint constraints^16,70^or STA models^11,77^.

An application which would benefit from our marker projection schemes is the pre-operative planning and criterion determination for performing femoral derotation osteotomies in children with CP, due to the significant reduction in joint angle errors and improved quality of computed kinematics in hip rotations - internal/external hip rotation - which we observed and which are leveraged in pre-operative planning and criterion determination for femoral osteotomies. In addition, the reduction in joint angle errors and increase in correlation with ground-truth kinematics were obtained for a wide variety of participants performing various tasks, thereby underscoring the potential the clinical usability of both our proposed marker projection schemes.

### Feasibility of microwave imaging

As discussed above, our results indicate that our marker-projection schemes (wherein the markers are projected onto the bone surface) both reduce joint angle errors and improve the quality of computed kinematics, specifically in hip rotations affected most by STA. However, in order to project the markers onto the bone surface, determining the location of the bone from the skin surface during dynamic motion is critical.

As discussed in the introduction, two notable studies proposed leveraging ultrasound imaging to locate the underlying bone^13,20,78^. However, incorporating ultrasound imaging in biomechanics has the following drawbacks: the need for a probe to be held at the location to be imaged, the requirement for coupling liquid to improve image resolution, and the prerequisite knowledge of reading ultrasound images (either through radiologist’s input or prior experience with ultrasound images) to clearly discriminate between bone and soft tissues.

Our results indicate that microwave imaging can be effectively applied in biomechanics and may be an effective alternative to ultrasound imaging. Data were collected under specific conditions in our study—no coupling liquid and a lower number of antennas—to evaluate the efficacy of applying microwave imaging in biomechanical applications. The conditions were chosen to emulate a wearable system which would be capable of real-time data acquisition. Using imaging algorithms - which have predominantly been applied in breast and brain tumour detection - we have successfully located the femur (on the mid-thigh) in all anatomical models, using all imaging algorithms tested in this study. This is especially notable as the bone is considered to be an extended scatterer in muscle^79^with imaging algorithms typically applied to detect point-like or small scatterers^26,56,80^. Additionally, the applicability and generalisability of our results are underscored by our detection of the femur in anatomical models of varying BMI scores and genders.

For all images reconstructed using confocal imaging algorithms, we observed maximum localisation errors of less than 2cm, with both SMR and SCR showing the presence of a scatterer (femur is considered a scatterer in muscle due to the difference in electric properties between muscle and bone). The localisation error was in a similar range to those obtained for tumour detection^35,80^and bone imaging studies applying different imaging algorithms^36^. Additionally, similar to other studies comparing DAS and DMAS confocal imaging algorithms, we obtained less artefacts and smaller localisation errors with DMAS compared with DAS. Contrary to our initial assumption, localisation errors reduced with BMI scores, with the smallest localisation obtained for the Ella-30 model. We hypothesise that this may be attributed to a closer match between the calculated velocity (to time-shift the signal) to that of the actual velocity, as the permittivity of muscle is used to determine the velocity and the Ella-30 model has a higher proportion of muscle (by volume) compared with other models.

We also observed that the reconstructed images, obtained using confocal imaging algorithms, are not able to accurately represent the shape and size of the underlying bone, with the reconstructed bone shown at the muscle-bone interface. We hypothesise this is a limitation of confocal imaging, which uses a simplified calculation of speed to time-shift the signal, thereby it only focuses on the location of maximum scattering, which is ideal for small scatterers (such as tumours) but not for extended scatterers (such as bones). Despite this, the location of the scatterer is within 2cm of the actual bone centre, which is sufficient for the projection of markers.

In contrast, results obtained from MUSIC were not only able to successfully determine the location of the femur from the skin surface in all anatomical models, but were also able to obtain the general shape and size of the bone. Similar to confocal imaging algorithms, MUSIC has predominantly been applied for imaging small scatterers^24,81^;however^36^, leveraged a variation of MUSIC (interferometeric MUSIC) to qualitatively image the bone. Specifically, they immersed a pig shank (with muscle, fat and bone layers) in a coupling liquid alongside the antennas and reconstructed images at each frequency multiplied with each other to reduce image artefacts. The authors reported reconstruction errors (localisation errors) of 2.78cm and 4.23cm, with no SMR or SCR values reported.

In our investigation, we leveraged a variation of MUSIC (wideband MUSIC) wherein images reconstructed at each frequency were summed together to reduce image artefacts. We obtained a maximum localisation error of 2.85cm across the four anatomical models, with both SMR and SCR values indicating the presence of a scatterer. We observed increases in SMR values with BMI scores, similar to the trend observed in confocal imaging algorithms. However, localisation errors also increased with BMI scores, in contrary to confocal imaging algorithms. Higher SMR values with higher BMI scores may be attributed to a lower mean scattering value in regions outside the object of interest (the bone) due to an increase in volume of the region outside the bone. However, the increase in localisation error with BMI scores when using MUSIC may be due to the decrease in the magnitude of electric field values—a key component of the MUSIC algorithm—inside the body with higher BMI scores, as there would be greater losses with higher volumes of soft tissues.

We obtained similar results were obtained using Kirchhoff migration, with both the location and the shape and size of the bone successfully reconstructed in all anatomical models. Kirchhoff migration has been applied as an alternative imaging algorithm to MUSIC, with results indicating that Kirchhoff migration is a fast, stable and effective imaging technique for detecting both large and small scatterers^61^, although its efficacy does reduce for large scatterers. As applied above, Kirchhoff migration, has not previously been investigated for bone imaging.

Our results indicate that Kirchhoff migration produces images with smaller reconstruction errors than MUSIC and comparable reconstruction errors to confocal imaging, whilst also reconstructing the shape and size of the bone. Similar to MUSIC, localisation errors increased with BMI scores; however, SMR values were comparable across BMI scores. This difference could be attributed to the difference in algorithms between MUSIC and Kirchhoff migration, wherein MUSIC detects a scatterer based on projection of the scattering matrix onto the noise subspace, whilst Kirchhoff migration utilises the entire scattering matrix to determine the location of the scatterer.

Our results indicate that microwave imaging was able to detect the location of the bone from the skin surface in models mimicking a phase of the gait cycle. Validating the efficacy of microwave imaging in posed models was critical to evaluate the feasibility of microwave imaging for biomechanical applications. This is because, as soft tissue deforms during motion, the distance of the bone from the skin surface in a posed state would be different to that obtained at static poses. Therefore, we also tested microwave imaging on the Ella-22 and Ella-30 models, as different magnitudes of soft tissue deformations were obtained from the two models for the same pose, due to their differences in soft tissue volume. The reconstructed images obtained using both MUSIC and Kirchhoff migration clearly indicate the location of the bone, with localisation errors obtained using MUSIC and Kirchhoff migration less than 2.7cm. Neither of the confocal imaging algorithms, DAS or DMAS, were able to successfully reconstruct the bone, with their reconstructed images showing several artefacts. Whilst we cannot definitively conclude, we hypothesise that the efficacy of confocal imaging algorithms may be affected by the computed imaging domain, with the angled imaging domain obtained in posed models potentially affecting the back-scattering ability of confocal imaging algorithms.

Combining the results from both of our investigations, we have proposed a safe and cost-effective method to reduce joint angle errors - specifically on hip joint angles most affected by STA - which may have the capacity to be applied clinically. A potential prototype of our system would incorporate a circular ring of wearable antennas^31^ placed around the thigh with reflective markers (or cluster of markers) attached on the ring. The absolute position of the ring would be provided by the skin-mounted marker-based system, with the distance of the bone from the ring computed using microwave imaging. This would enable projection of the cluster of markers onto the bone surface to reduce the effects of STA.

Our study has a few limitations. The challenges faced in our study investigating the efficacy of our marker projection schemes were in the method used to determine the actual bone location and in the method employed to calculate the trajectories of projected markers. Whilst we endeavored to determine the actual bone location using informed approximations based on dual-fluoroscopy markers in the LCS, the location may not reflect real-world locations during motion. Secondly, the determination of the trajectories of projected markers was based on kinematics computed using inverse kinematic pipelines applied to dual-fluoroscopy data, and may therefore be affected by model constraints and the MKO method. Thus, future investigations comparing kinematics computed using our marker-projection schemes to actual bone movement may further underscore their potential to improve kinematics and reduce the impact of STA.

Another limitation of our investigation is that the data leveraged in our study only contains artefact-free bone movement and skin-mounted markers of the hip and thigh. Therefore, we have not been able to test the efficacy of our marker-projection schemes on other joints like the scapular joint, which is known to be affected by STA^82–84^. However, we have compared our marker-projection schemes to the one proposed for the scapular joint^15^, with our results indicating that our proposed schemes produce lower joint angle errors and improved correlation to that obtained from Begon-projected markers.

There were also notable limitations of the microwave imaging investigations we performed. Namely, the metrics utilised (SMR, SCR and localisation error), the hotspots generated in the reconstructed images, and emulating both a dynamic pose for the ViP models and a wearable system. The metrics leveraged in analysing the feasibility of microwave imaging were predominantly created for small scatterers, such as breast tumours, which have a higher permittivity than the surrounding medium. Whereas, in our investigation, the object of interest (the bone) is an extended target and is of a lower permittivity to that of the background medium. Additionally, the imaging domain is made of heterogeneous layers - skin, fat,muscle and bone - resulting in scattering at various boundaries. The above reasons may have contributed to the negative SCR values and varying SMR values. Whilst positive SCR scores have been used as an indicator of tumours in breast imaging studies^65^, reported negative SCR values for heterogeneous and denser breasts.

Similarly, localisation error has predominantly been applied for small scatterers. Localisation error is calculated as the distance between the location of the maximum intensity in the image to that of the expected location of the scatterer. Therefore, localisation errors can be affected by large hotspots, wherein the maximum intensity may not be at the centre of the hotspot or the maximum intensity may be obtained at a different hotspot than that of the object of interest. The limitation of leveraging localisation errors for extended targets is exemplified by the relatively high values of 2cm (compared to magnitudes of STA) we obtained, despite an overlap between the actual bone location and the hotspot representing the bone. These relatively large localisation errors may be misleading, as only the detection of the bone edge is required for projection of markers, and could be misinterpreted as indicating that microwave imaging cannot be leveraged to project markers and subsequently reduce STA.

The spread of hotspots in the reconstructed images obtained using MUSIC and Kirchhoff migration may also be attributed to scattering obtained at multiple interfaces. Additionally, hotspots at locations different to that of the bone may be caused by scatterinat fat/muscle interfaces, or by scattering from muscle/blood vessel interfaces. These interfaces are more pronounced in images reconstructed using MUSIC and Kirchhoff migration than images obtained using confocal imaging algorithms, as confocal imaging algorithms do not leverage electric field values to generate images.

Whilst one of the primary goals was to undertake an exploratory study to assess the viability of applying microwave imaging in biomechanical applications, with data collected under conditions aimed to emulate a wearable system, major challenges remain in the application of microwave imaging in dynamic situations. Firstly, the antennas on the posed ViP models were moved based on the deformations the soft-tissues undergo during movement and not just the movement of the underlying bone. This was to reflect and emulate the movement of skin-mounted sensors as close as possible, as skin-mounted sensors would be affected by the deformations of soft-tissues. Additionally, the relative location of the antennas with respect to one another was kept constant. Whilst the latter could be achieved by the development of circular antenna arrays^31^, the physics-based deformations of the ViP models may not account for additional inhomogenity of soft-tissues and soft-tissue deformations encountered during actual motion of the human body. Secondly, the data generated for investigating microwave imaging in this study do not account for sensor cross-talk and additional variabilities which antennas may encounter in real-world applications. This could be alleviated by complementing the simulations with issues encountered by studies developing wearable systems incorporating multiple antennas^30,32^. Thirdly, the heating effects of the antennas were not analysed; however, any potential heating effect could be ameliorated by incorporating antennas designed to be used in direct contact with the skin whilst emitting lesser power than a conventional cellphone^85^. Lastly, the decision to incorporate a small number of antennas was to ensure that the potential time taken for data acquisition was smaller than the time taken for movement, so that the soft-tissues encompassed by the antennas may be considered static for the duration of data acquisition. Whilst this was assumed in this study, leveraging radar-on-chip devices^31^, time-domain recordings^30^and no mechanical movement of antennas^86^, can help in reducing the time taken for data acquisition in real-world systems.

In conclusion, we have proposed a novel and generalisable method to reduce the deleterious impact of STA on computed kinematics, specifically on the hip, which can be applied for a range of motions and participants. Our proposed marker projection schemes reduce kinematic errors significantly when compared with conventional (un-projected) markers and outperform other marker projection schemes. Additionally, they improve the quality of computed kinematics, as evidenced by higher correlation values. We have also shown that microwave imaging—a safe, cost-effective and operator-independent imaging modality not previously applied in biomechanics—can be used in biomechanical applications. Through the use of wearable antennas and the collection of data under specific conditions, our results indicate that the femur can be successfully located from the skin surface (enabling marker projection) for anatomical models of varying BMI scores and genders, and in poses mimicking standing and a phase of the gait cycle. Therefore, we have proposed and validated an end-to-end multi-disciplinary solution which we hope can be applied in clinical gait analysis to reduce the impact of STA on computed kinematics.

## Data Availability

Data available upon reasonable request from the corresponding author
